# Antibody drug conjugates in the clinic

**DOI:** 10.1002/btm2.10677

**Published:** 2024-05-04

**Authors:** Edidiong Udofa, Disha Sankholkar, Samir Mitragotri, Zongmin Zhao

**Affiliations:** ^1^ Department of Pharmaceutical Sciences University of Illinois Chicago Chicago Illinois USA; ^2^ University of Michigan Ann Arbor Ann Arbor Michigan USA; ^3^ John A. Paulson School of Engineering and Applied Sciences Harvard University Cambridge Massachusetts USA; ^4^ Wyss Institute for Biologically Inspired Engineering at Harvard University Boston Massachusetts USA; ^5^ University of Illinois Cancer Center Chicago Illinois USA

**Keywords:** ADC, antibody, antibody–drug conjugate, cancer, cancer treatment, chemotherapy, clinic, clinical translation, clinical trial, drug delivery, FDA

## Abstract

Antibody‐drug conjugates (ADCs), chemotherapeutic agents conjugated to an antibody to enhance their targeted delivery to tumors, represent a significant advancement in cancer therapy. ADCs combine the precise targeting capabilities of antibodies and the potent cell‐killing effects of chemotherapy, allowing for enhanced cytotoxicity to tumors while minimizing damage to healthy tissues. Here, we provide an overview of the current clinical landscape of ADCs, highlighting 11 U.S. Food and Drug Administration (FDA)‐approved products and discussing over 500 active clinical trials investigating newer ADCs. We also discuss some key challenges associated with the clinical translation of ADCs and highlight emerging strategies to overcome these hurdles. Our discussions will provide useful guidelines for the future development of safer and more effective ADCs for a broader range of indications.

**Translational Impact Statement**

This review aims to provide an overview of the current clinical landscape of antibody‐drug conjugates (ADCs), an emerging modality for targeted cancer therapy. We discuss Food and Drug Administration‐approved ADC products and highlight the diversity of new investigative ADCs in active clinical trials based on their indication, antibody type, target antigen, and payload while also outlining the challenges in ADC development. Together, this review provides an understanding of the current state of ADCs in the clinic while fostering research initiatives to improve ADC development.

## 1 INTRODUCTION

Cancer has long been a global challenge, recognized as the second leading cause of death worldwide, accounting for one in six deaths.^1^, ^2^ Traditional treatment methods—surgery, radiotherapy, and chemotherapy—have been the cornerstone of cancer management for decades. However, their effectiveness is hampered by several factors such as the stage of cancer at diagnosis (limiting surgery's viability), the damage to healthy cells, organs, and tissues (a consequence of radiotherapy and chemotherapy), and the development of drug resistance (a challenge for chemotherapy).^1^, ^2^ Moreover, these treatments tend to focus on the cancer's location or histological features rather than on specific molecular changes.^3^ Recent advances in molecular and tumor biology have shifted cancer treatment from these broad approaches to more personalized and precise therapies.^3^ Inspired by Paul Ehrlich's “magic bullet” concept, new cancer treatment options aim to minimize toxicity by targeting specific molecular markers of cancer. Targeted therapies, which include monoclonal antibodies (mAbs) and small‐molecule inhibitors, have transformed the management of various cancers, such as those affecting the breast, colon, lungs, and digestive tract, enhancing the efficacy of traditional chemotherapy.^4^, ^5^, ^6^

The foundation for antibody‐based therapies was laid in the 1960s with the identification of tumor antigen expression and the development of antibodies in the late 19th century. mAbs have proven effective in both diagnosing and treating hematological malignancies and solid tumors.^7^, ^8^ They work by targeting tumor‐associated antigens, either inhibiting cell growth and angiogenesis or stimulating a long‐lasting immune response against the tumor.^9^, ^10^ This led to the creation of Antibody‐Drug Conjugates (ADCs), which merge the targeted approach of mAbs with the cell‐killing power of chemotherapy, sparing healthy tissue and thus representing a significant advancement in cancer therapy.^11^, ^12^, ^13^ Over the past few decades, clinical studies of ADCs have been increasingly active. To date, the US Food and Drug Administration (FDA) has approved 11 ADCs, with two additional approvals by other regulatory agencies. Numerous ADCs are under clinical investigation, promising to expand the range of treatable cancers. Ongoing trials are also exploring the most effective treatment combinations using approved ADCs. In this review, we provide an overview of the clinical landscape of ADCs. We discuss the design considerations and mechanism of actions of ADCs, highlight approved products, and review >500 active clinical trials involving both approved and new investigative ADCs. We also discuss the challenges for clinical translation of ADCs and provide a prospect for the future development of more effective and safer ADCs.

## 2 KEY COMPONENTS AND MECHANISM OF ACTIONS OF ADCs

### 2.1 Key components

An ADC is composed of an antibody conjugated to the cytotoxic payload by a chemically stable linker. While this sounds simple, the complexity of the ideal properties of each of these components has impacted the progress of ADC research.^14^ Here, we discuss key considerations related to the design of each ADC component.

#### 2.1.1 Antibody

mAbs, which have specificity to a particular antigenic epitope, are more commonly used to formulate ADCs.^15^, ^16^ The antibody can be considered the driver that facilitates the specific delivery of the payload to tumor cells. The generally recommended properties of the antibody component include: (i) high selectivity for cancer antigens over healthy cells, and (ii) high target binding affinity.^15^ Other desirable properties include strong retention after binding, low immunogenicity, and minimal cross‐reactivity. Earlier generations of ADCs were formulated using murine mAbs, which were problematic due to immunogenicity that reduced efficacy. However, newer generations of ADCs employ humanized antibodies, which have a lower risk of immune activation.^14^, ^15^

A key aspect in designing the antibody component is the selection of antigenic targets. Ideally, the target should be exclusively expressed on tumor cells.^15^, ^17^, ^18^ However, a more realistic goal is to identify a target that (i) has high expression on tumor cells and low expression on healthy cells, with a minimum target antigen threshold of >10,000 copies/cell,^18^, ^19^ (ii) is displayed on the surface of tumor cells with minimal shedding to enable efficient antibody binding, and (iii) has the ability to be internalized to aid the transport of ADC into the cell.^14^, ^15^, ^17^, ^20^ There are over 50 known antigens used in ADCs, and common antigens in approved ADC products include HER2, Trop2, B‐cell maturation antigen (BCMA), Nectin4, CD19, CD22, CD30, CD33, and CD79b.^15^ More recently, research focus has also shifted to the identification of antigens beyond the tumor cells. Antigens expressed in the tumor microenvironment, such as on the stroma, vasculature, extracellular matrix, and tumor matrix, have the potential to broaden the target antigen scope of ADCs. Additionally, antigens expressed in these areas are less susceptible to mutations and could prevent the development of drug resistance.^17^

The size of the antibody in an ADC is also important.^17^ Immunoglobulin G (IgG) antibodies (IgG1, IgG2, IgG3, IgG4) are commonly used in ADCs.^15^ IgG1 is the most commonly employed subtype due to its abundance in the serum and strong effector functions, while IgG3 is rarely used due to its short half‐life in the blood.^18^ While IgG antibodies are the most common in the serum, their large size often limits penetration through the blood capillaries and tumor tissue. To overcome this, newer ADCs are formed with miniaturized antibodies by removing the fragment crystallizable (Fc) segment. This has made ADCs more applicable to solid tumors but also comes with the problem of reduced half‐life.^17^

In the design of ADCs, a careful balance between the antibody's binding affinity and internalization is important. Often, higher binding affinity results in rapid internalization of the antibody. However, in the case for solid tumors, the rapid internalization of ADCs mostly occurs at the tumor periphery only.^17^, ^19^ This effect is because of the binding site barrier, which causes the trapping of ADCs near the blood vessels in solid tumors hindering their penetration to distant tumor cells.^17^, ^21^

Aside from enabling delivery of the payload, the antibody also plays some cytotoxic functions such as antibody‐dependent cytotoxicity (ADCC), antibody‐dependent cellular phagocytosis (ADCP), and complement‐dependent cytotoxicity (CDC),^14^ which will be further discussed in a later section.

#### 2.1.2 Linker

The linker is an important component that influences the stability, payload release, pharmacokinetics (PK), toxicity, and overall therapeutic efficacy of ADCs.^14^, ^22^, ^23^ Most recent advances in ADCs are due to improvements in drug‐linker technologies.^22^ An ideal ADC linker should be stable enough in circulation to prevent premature drug release while also being sufficiently sensitive to the release stimuli at the target site.^19^, ^24^, ^25^, ^26^

ADC Linkers are broadly classified as either cleavable or noncleavable. Cleavable linkers can be chemical cleavage linkers (such as hydrazone or disulfide bond based) or enzyme cleavage linkers (such as glucuronide or peptide bond based).^17^, ^19^, ^24^ Upon internalization of ADCs into target cells, such linkers are degraded through several mechanisms such as proton lysis, thiol reduction, proteolysis, or carbohydrate hydrolysis, resulting in the release of the cytotoxic payload.^24^ This cleavage occurs in the endosomal‐lysosomal compartment of the tumor cells. Because the stimuli responsible for the cleavage of these linkers are not exclusively restricted to tumor cells, some of these linkers are also susceptible to chemicals and enzymes in the blood or tumor microenvironment, increasing the risk of systemic toxicity.^19^

Conversely, noncleavable linkers enable to release the payload by enzymatic degradation of the antibody in the endosome/lysosome.^27^ The linker remains conjugated to the payload along with some amino acid residues, which restricts the diffusion of the payload across the cells, thus reducing systemic toxicity.^18^, ^26^ These linkers are less susceptible to the physiological environment, resulting in increased plasma stability^16^, ^17^ and specific drug release.^27^ However, the persistence of the linker and amino acid residue could affect the function of the payload; hence, only small molecules that tolerate chemical modifications are suitable for these linkers.^17^, ^18^

Other considerations for the ADC linker include its length and hydrophobicity. Accumulating evidence indicates that shorter linkers improve the stability of ADCs as the payloads benefit from the steric shield provided by the antibody.^23^ Simultaneously, hydrophilic linkers increase the solubility and improve the PK of ADCs and are more beneficial for ADCs with hydrophobic payloads.^19^, ^22^, ^25^

#### 2.1.3 Payload

ADCs are formulated with highly potent payloads that possess picomolar or nanomolar IC_50_ to ensure cytotoxic efficacy.^15^ Earlier generations of ADCs utilized conventional chemotherapeutic drugs; however, due to the limited amount (1%–2%) of the payload reaching the target site, the efficacy of these moderately potent agents was suboptimal.^14^, ^17^, ^19^ The new generation of approved ADCs deploys more potent payloads that inhibit microtubules necessary for cell division or inflict damage on cell DNA. The potency of these agents exceeds that of traditional chemotherapy by more than 100‐ to 1000‐fold.^18^, ^21^, ^28^ Examples of such microtubule‐targeting agents include Dolastatin10‐based auristatin analogs and maytansinoids, and commonly used DNA‐damaging payloads include Calicheamin analogs (inducing DNA double‐strand breaks), Duocarmycin analogs (promoting DNA alkylation), and topoisomerase 1 inhibitors (causing DNA intercalation).^18^ Having an intracellular target is an important requirement for these payloads, as they are designed to be released within the tumor cells.^28^ A thorough review of ADC payloads can be found in recent reviews published elsewhere.^21^, ^28^, ^29^

Beyond their high potency, ADC payloads should also exhibit several other key properties including stability in systemic circulation, resistance to degradation within endosomes/lysosomes, minimal immunogenicity, a relatively low molecular weight, and chemical groups amendable to conjugation with the linker. Additionally, an appropriate hydrophobicity of the payload is needed to balance solubility for successful conjugation to the ADC with good cellular permeability while preventing rapid clearance.^15^, ^17^, ^19^, ^28^

### 2.2 Mechanisms of action

**FIGURE 1:**
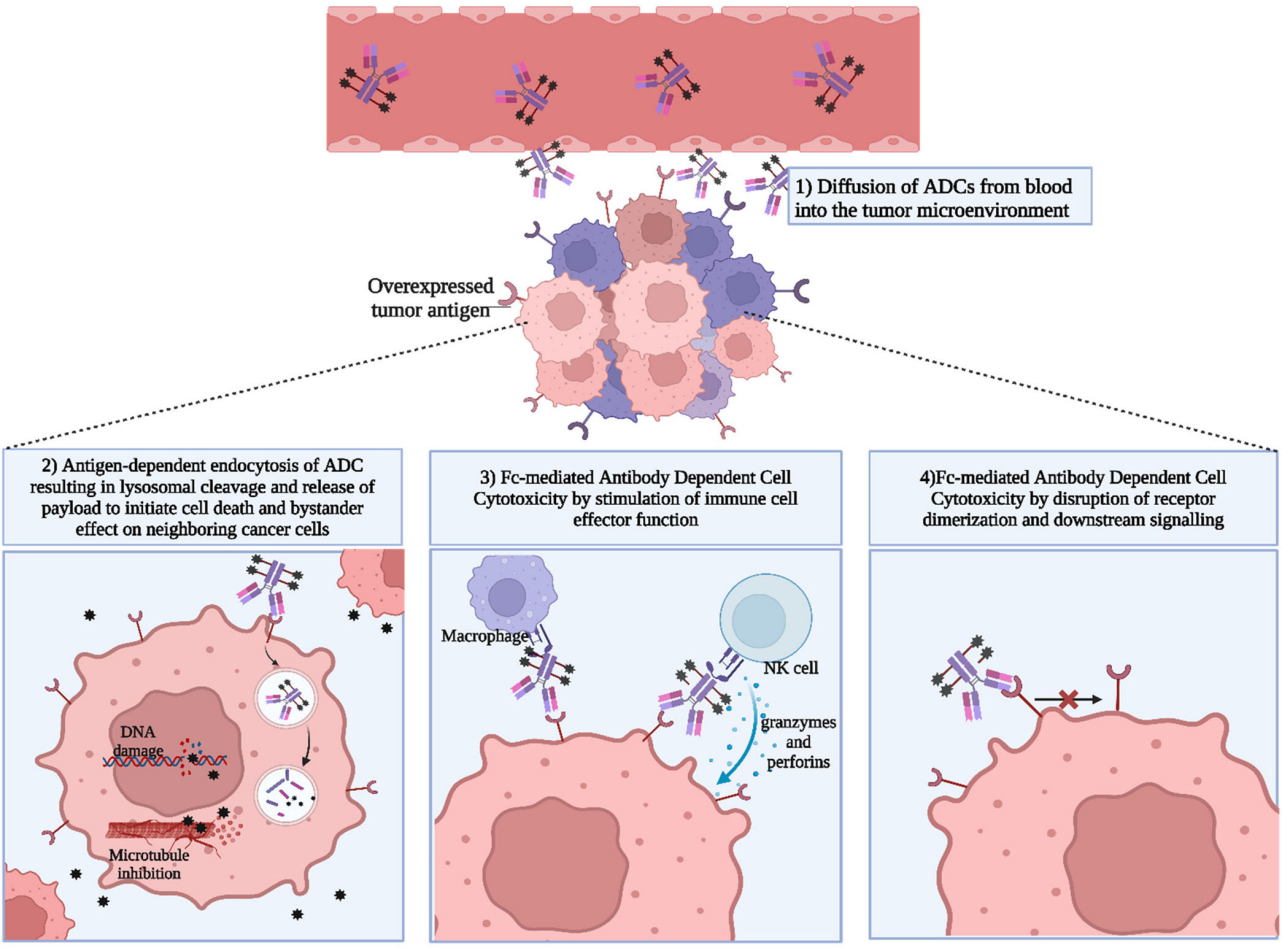
Schematic representation of the mechanisms of action of antibody‐drug conjugates (ADCs). Created with BioRender. Fc, fragment crystallizable.

Figure 1 provides an overview of the mechanism of actions of ADCs. After intravenous administration, ADCs are distributed throughout the body and accumulate in the tumors. The circulation of ADCs is facilitated by the long half‐life of the antibody component, while their accumulation within tumor is driven by the binding of the fragment antigen‐binding (Fab) segment of the antibody to the antigenic target.^30^ The large size of mAbs limits the diffusion of ADCs through tumor vasculature, resulting in only a small fraction (0.0003%–0.08% per gram of tumor^31^) of the administered dose eventually accumulating at the target site, underscoring the need for a highly potent payload.^32^ The binding of the antibody to its target triggers the internalization of the ADC, which can occur via clathrin‐mediated endocytosis (CME), caveolar‐mediated endocytosis, or pinocytosis.^15^, ^27^ Subsequently, the ADC is packed into an early endosome, which matures and fuses with a lysosome where the payload is released upon endosomal/lysosomal degradation of the ADC. The type of linker determines the payload release mechanism postinternalization.^32^ Noncleavable linkers require ADC localization in the lysosome for proteolytic degradation, whereas the payload release from cleavable linkers is triggered by intracellular stimuli (such as pH sensitivity, protease sensitivity, or glutathione sensitivity), bypassing the need for lysosomal trafficking.^27^, ^33^

Once the payload is released into the cytoplasm, it disrupts cellular functions through microtubule inhibition or DNA damage, leading to cancer cell death. This is the primary mechanism of action of ADCs. In addition to enhanced payload delivery to targeted cells, some ADCs can display bystander‐killing effect where the released payload permeates through the cell membrane, diffusing to and killing neighboring tumor cells. Moreover, CDC, ADCC, and ADCP are additional mechanisms to enhance ADC's effect. These mechanisms are medicated by the interaction of the Fc region of the antibody with the neonatal Fc receptors present on effector immune cells.^34^ In ADCC, the Fc region's interaction with the Fc_Ɣ_ receptor activates immune cells like natural killer (NK) cells, which release cytotoxic molecules (e.g., perforins and granzymes). ADCP involves macrophages engulfing cancer cells following a similar interaction.^9^ However, these Fc‐mediated actions can also reduce ADC efficacy by causing ADCs to be sequestered by immune cells, leading to off‐target toxicity.^35^ However, these effector functions are mainly associated with IgG1, as IgG2 and IgG4 are less effective in medicating Fc‐dependent activities.^18^, ^34^ Furthermore, another mechanism of action of ADC involves its inhibition of downstream signaling pathways. This is particularly observed with antigenic targets upstream of the oncogenic pathway, where antibody binding prevents the dimerization of the receptors.^32^, ^34^

## 3 FDA‐APPROVED ADC PRODUCTS

Currently, there are 11 FDA‐approved ADC products and two more approved by other regulatory agencies (NMPA and PMDA). Another two ADCs (Moxetumomab pasudotox and Belantamab mafodotin‐blmf) were previously approved by the FDA but were withdrawn due to limited clinical use or failure to meet primary efficacy endpoints. All the approved products are administered intravenously and are intended to treat a specific type of cancer. Key information for each approved product is summarized in Table 1.

**TABLE 1:** Information on ADC products approved by the FDA or other regulatory agencies.

	Name	Payload (payload class), payload action	Linker type	Target antigen	Monoclonal antibody isotype	Approved indication	Approval year
Hematological tumors	Gemtuzumab ozogamicin/Mylotarg® (Pfizer/Wyeth)	Ozogamicin (Calicheamicin), DNA cleavage	Acid cleavable	CD33	IgG4	Relapsed acute myelogenous leukemia	FDA (2000, 2017)
	Brentuximab vedotin/ADCETRIS® (Seattle Genetics)	MMAF (Auristatin), Microtubule inhibitor	Cleavable	CD30	IgG1	Relapsed and/or refractory systemic anaplastic large cell lymphoma (2011); Relapsed and/or refractory primary cutaneous anaplastic large cell lymphoma or CD30+ mycosis fungoides (2017); Classical Hodgkin lymphoma, systemic anaplastic large cell lymphoma, or CD30+ peripheral T‐cell lymphoma (2018)	FDA (2011, 2017, 2018); EMA (2012)
	Inotuzumab ozogamicin/Besponsa® (Pfizer/Wyeth)	Ozogamicin (Calicheamicin), DNA cleavage	Acid cleavable	CD22	IgG4	Relapsed or refractory B‐cell precursor acute lymphoblastic leukemia	FDA (2017)
	Moxetumomab pasudotox/Lumoxiti® (Astrazeneca)	Pseudomonas exotoxin (Pseudotox), Inhibitor of protein synthesis	Recombinant covalently fused (linkerless)	CD22	IgG4	Relapsed or refractory hairy cell leukemia	FDA (2018)‐ withdrawn
	Polatuzumab vedotin‐piiq/Polivy® (Genentech, Roche)	MMAE (Auristatin), Microtubule inhibitor	Enzyme cleavable	CD79b	IgG1	Relapsed or refractory diffuse large B‐cell lymphoma	FDA (2019)
	Belantamab mafodotin‐blmf/Blenrep® (GlaxoSmithKline (GSK))	MMAF (Auristatin), Microtubule inhibitor	Noncleavable	BCMA	IgG1	Relapsed or refractory multiple myeloma	FDA (2020)‐withdrawn
	Loncastuximab tesirine‐lpyl/Zynlonta® (ADC Therapeutics)	SG3199 (PBD Dimer), DNA cleavage	Enzyme cleavable	CD19	IgG1	Relapsed or refractory diffuse large B‐cell lymphoma	FDA (2021)
Solid tumors	Ado‐trastuzumab emtansine/Kadcyla® (Genentech, Roche)	DM1 (Maytansinoid), Microtubule inhibitor	Noncleavable	HER2	IgG1	HER2+ metastatic breast cancer previously treated with trastuzumab and a taxane (2013); HER2+ early breast cancer after neoadjuvant taxane & trastuzumab‐based treatment (2019)	FDA (2013, 2019)
	Fam‐trastuzumab deruxtecan‐nxki/Enhertu® (AstraZeneca/Daiichi Sankyo)	DXd (Camptothecin), TOP1 inhibitor	Enzyme cleavable	HER2	IgG1	Unresectable or metastatic HER2+ breast cancer after 2 or more anti‐HER2 regimens (2019) d; locally advanced or metastatic HER2+ gastric or gastroesophageal junction adenocarcinoma after a trastuzumab‐based regimen (2021)	FDA (2019, 2021)
	Enfortumab vedotin‐ejfv/Padcev® (Astellas/Seattle Genetics)	MMAE (Auristatin), Microtubule inhibitor	Enzyme cleavable	Nectin4	IgG1	Locally advanced or metastatic urothelial cancer after a PD‐1 or PD‐L1 inhibitor and a Pt‐containing chemotherapy (2019) or are ineligible for cisplatin‐containing chemotherapy and previously received 1 or more lines of therapy (2021)	FDA (2019, 2021,2023)
	Sacituzumab govitecan‐hziy/Trodelvy® (Immunomedics)	SN‐38	Acid cleavable	TROP2	IgG1	Locally advanced or metastatic triple‐negative breast cancer; locally advanced or metastatic urothelial cancer	FDA (2020, 2021)
	Tisotumab vedotin‐tftv/Tivdak® (Seagen Inc)	MMAE (Auristatin), Microtubule inhibitor	Enzyme cleavable	Tissue Factor (TF)	IgG1	Recurrent or metastatic cervical cancer	FDA (2021)
	Mirvetuximab soravtansine/ELAHERE™ (ImmunoGen)	DM4 (Maytansinoid), Microtubule inhibitor	Cleavable	FRα	Undisclosed	Ovarian cancer, fallopian tube cancer, primary peritoneal cancer	FDA (2022)
	Cetuximab sarotalocan/Akalux® (Rakuten Medical)	IRDye700DX, photoimmunotherapy	None	EGFR	IgG1	Unresectable locally advanced or recurrent HNSCC	PMDA (2020)
	Disitamab vedotin/Aidixi® (RemeGen)	MMAE (Auristatin), Microtubule inhibitor	Enzyme cleavable	HER2	IgG1	Locally advanced or metastatic gastric cancer	NMPA (2021)

Abbreviations: ADC, antibody‐drug conjugates; FDA, Food and Drug Administration; IgG, Immunoglobulin G; MMAE, monomethyl auristatin E; MMAF, monomethyl auristatin F.

### 3.1 Approved ADCs for hematological cancers

To date, seven ADCs have been approved by the FDA for treating hematological cancers, with two of them having been withdrawn due to limited clinical efficacy or use. Mylotarg® (Gemtuzumab Ozogamicin, GO) is the first ADC approved for the treatment of acute myelogenous leukemia (AML), characterized by poor bone hematopoiesis.^36^ GO was initially approved in 2000 for AML but was then voluntarily withdrawn from the market because of failing to demonstrate clinical benefits and excessive fatal toxicities. Before the approval of GO, the standard of care for AML was a 7 + 3 regimen, involving a 7‐day treatment with cytarabine followed by a 3‐day treatment with daunorubicin.^36^, ^37^ GO was reapproved in 2017 with new data showing safety and efficacy after dose adjustment for CD33+ AML in patients of 2 years and older.^38^, ^39^ It consists of a humanized anti‐CD33 IgG4 mAb linked to DNA‐damaging calicheamicin via a covalent linker. The linker is acid‐cleavable and enables the release of the payload in the endosome/lysosome of myeloblasts. The treatment is not considered intensive and hence is suitable for elderly patients and patients with comorbidities.^37^ In the Phase 3 trial of 280 patients in France (ALFA‐0701 Trial), GO demonstrated superior event‐free survival for patients with newly diagnosed AML,^39^ and a meta‐analysis of 3325 adult patients also showed an overall improvement in survival.^38^ However, Mylotarg® comes with a blackbox warning for hepatotoxicity, and other warnings include the risk of severe hemorrhage and infusion‐related reactions.

ADCETRIS® (Brentuximab vedotin) is the standard of care for treating patients with refractory or relapsed classical Hodgkin Lymphoma (cHL). It was most recently approved by the FDA in 2018 and consists of a human chimeric anti‐CD30 IgG1 antibody covalently linked with monomethyl auristatin E (MMAE) via a valine‐citrulline cleavable linker.^40^ Upon binding to and internalization by CD30+ cancer cells, the linker undergoes cleavage by endosomal/lysosomal proteases to release MMAE, which induces cell death via apoptosis.^41^ Brentuximab vedotin has also been reported to be active in diseases with low CD30 expression, due to its bystander effect where free MMAE diffuses to and kills adjacent cancer cells.^41^, ^42^ Before its approval, the frontline treatment of cHL involved a combination of chemotherapy agents: doxorubicin, bleomycin, vinblastine, and dacarbazine, but this treatment regimen is associated with relapse in up to 40% of patients. The Phase 3 ECHELON‐1 trial revealed that replacing bleomycin with brentuximab vedotin in this treatment regimen led to superior progression‐free survival in patients with stage III and IV cHL,^43^ and long‐term follow‐up showed that this benefit is sustained.^41^, ^44^ However, ADCETRIS® comes with a blackbox warning for progressive multifocal leukoencephalopathy.^45^

Besponsa® (Inotuzumab ozogamicin) is the only ADC approved for the treatment of relapsed or refractory B‐cell precursor acute lymphoblastic leukemia (R/R ALL). It is a humanized anti‐CD22 IgG4 antibody covalently conjugated to calicheamicin via a butanoic acid liable linker.^46^, ^47^ In this case, the cytotoxicity is mediated only by the payload upon release, and its effectiveness is thus dependent on effective internalization and sensitivity to calicheamicin.^46^ Inotuzumab ozogamicin has been reported to improve clinical outcomes compared with salvage chemotherapy.^48^ The INO‐VATE Phase 3 trial revealed that Inotuzumab ozogamicin led to a higher response rate than standard‐of‐care chemotherapy.^46^, ^49^ The overall response rate of Inotuzumab ozogamicin is 60%–80% in patients with R/R ALL.^47^

Polivy® (Polatuzumab vedotin‐piiq) received accelerated FDA approval in 2019 for the treatment of R/R diffuse large B‐cell lymphoma (DLBCL) in combination with bendamustine and rituximab (BR).^50^ The Phase 2 trials of Polatuzumab vedotin‐piiq demonstrated a higher complete response (CR) rate and reduced the risk of death in patients with transplantation‐ineligible R/R DLBCL by 58% in patients treated with a combination of Polatuzumab vedotin with BR compared with BR alone.^51^ Polatuzumab vedotin consists of a humanized anti‐CD79b IgG1 mAb linked to MMAE via a protease cleavable linker. This formulation uses an engineered cysteine (THIOMABs) to achieve the efficient and homogenous conjugation of antibody with MMAE.^52^

Zynlonta® (Loncastuximab tesirine, SG3199), consisting of a humanized anti‐CD19 IgG1 antibody conjugated to a pyrrolobenzodiazepine (PDB) dimer cytotoxin, is another approved ADC for the treatment of DLBCL. Upon endosomal/lysosomal cleavage, SG3199 forms inter‐strand crosslinks within the cell's DNA leading to cell death. SG3199 also exhibits a bystander‐killing effect.^53^, ^54^ In July 2023, further clinical trials on Zynlonta® were terminated due to FDA's hold on this ADC, stemming from concerns over excessive fatal toxicities.

LUMOXITI® (moxetumomab pasudotox‐tdfk) was initially approved in 2018 for the treatment of adult patients with R/R hairy cell leukemia. This ADC comprises the Fv fragment of a CD22‐targeting antibody conjugated to an immunotoxin. Once released, the immunotoxin induces apoptosis through the catalysis of ADP‐ribosylation of the diphthamide residue in elongation factor‐2. LUMOXITI's approval was based on a Phase 3 study that showed up to 90% of circulating CD19+ B cells were depleted by Day 8 of treatment.^55^ Although this study reported a generally acceptable tolerability profile, LUMOXITI was withdrawn from the market in 2023 due to inadequate clinical use.

Blenrep® (Belantamab mafodotin‐blmf) was approved in 2020 for the treatment of R/R multiple myeloma in adult patients.^56^ Belantamab mafodotin‐blmf was a first‐in‐class ADC with an anti‐BCMA antibody and the first ADC with the microtubule inhibitor, monomethyl auristatin F (MMAF) payload to receive approval. Its approval was based on the DREAMM‐2 global trial, which demonstrated an overall response rate of 31%. However, 77% of patients receiving the treatment of Belantamab mafodotin‐blmf experienced ocular toxicity, leading to a black‐box label by the FDA on this product.^57^ In 2020, GSK announced the withdrawal of Blenrep® from the US market as it failed to meet the primary endpoint in the DREAMM‐3 confirmatory clinical trial.^58^

### 3.2 Approved ADCs for solid tumors

To date, six ADCs have been approved by the FDA and two more by other regulatory agencies for treating solid tumors. Kadcyla® (Ado‐trastuzumab emtansine) is the first ADC approved by the FDA for the treatment of HER2‐positive metastatic breast cancer.^59^ Ado‐trastuzumab emtansine (T‐DM1) consists of a humanized anti‐HER2 IgG1 antibody known as trastuzumab, which was introduced in 1998 for the treatment of HER2+ breast cancers. Up to 25% of breast cancer patients exhibit HER2 overexpression, which is associated with poor prognosis.^60^, ^61^ However, a significant portion of patients under trastuzumab treatment did not respond or experienced relapse. Ado‐trastuzumab emtansine is a combination of trastuzumab and the microtubule‐inhibiting maytansinoid, linked via a nonreducible thioether linker. Endosomal/lysosomal degradation of the antibody leads to the release of maytansinoid causing apoptosis. There is also additional antibody‐mediated cytotoxicity due to the downregulation of HER2, inhibition of HER2 dimerization, activation of immune response, and ADCC.^61^, ^62^ Various clinical studies have shown improvement in overall survival and quality of life in patients treated with Ado‐trastuzumab emtansine.^59^, ^63^ Despite the improvement in outcome noted with T‐DM1, there are concerns about the development of drug resistance observed in initial responders.^62^ Kadcyla® also comes with FDA‐boxed warnings for hepatotoxicity, cardiotoxicity, pulmonary toxicity, and embryo‐fetal toxicity.^64^

Padcev® (Enfortumab vedotin‐ejfv) is a first‐in‐class ADC for the treatment of metastatic urothelial carcinoma (UC), an aggressive cancer with a poor prognosis. Enfortumab vedotin‐ejfv (EV) consists of a fully humanized anti‐Nectin‐4 IgG1 antibody linked to MMAE.^65^ Enfortumab vedotin‐ejfv received accelerated FDA approval in 2019 based on the Phase 1 and 2 trials indicating that EV had a high response rate, disease control rate, and improved overall survival in UC patients. The Phase 2 (EV‐201) trial, in which 90% of enrolled patients had metastatic visceral disease, showed that EV led to an overall response rate of 44% and complete remission rate of 12%. A Phase 3 (EV‐301) trial with 608 patients demonstrated the superior efficacy of EV compared with single‐agent chemotherapy.^66^, ^67^ This facilitated the approval of EV in 2021 by the FDA for the treatment of UC in two adult populations, including patients who had previously received a PD‐L1 inhibitor and platinum‐based chemotherapy and patients who are ineligible for cisplatin‐based chemotherapy.^67^ Padcev® comes with an FDA‐boxed warning for serious skin reactions.

Enhertu® (Fam‐trastuzumab deruxtecan‐nxki, T‐DXd) is the first ADC approved for HER2‐low breast cancer, accounting for about 40%–50% of HER2‐negative breast cancers. T‐DXd is composed of a humanized anti‐HER2 IgG1 linked to a topoisomerase‐1 inhibiting exatecan derivative (DXd) via a stable tetrapeptide linker. Like trastuzumab emtansine, the payload is released within the cancer cell. However, with a drug‐to‐antibody ratio (DAR) of 8, T‐DXd led to an efficient delivery of DXd even to tumors with low HER2‐expression.^68^, ^69^ T‐DXd was granted accelerated approval in 2019 for the treatment of patients with unresectable or metastatic breast cancer. This expansion of indication to cover all HER2‐expressing tumors allows for flexibility in the use of the medication. Its initial approval was based on a Phase 2 trial indicating an overall response rate of 60.3%.^68^ Following a Phase 3 trial indicating meaningful improvement in progression‐free survival and overall survival, T‐DXd received regular approval from the FDA in 2022.^69^, ^70^ Based on the DESTINY‐Gastric01, TXd was also approved in 2021 for the treatment of locally advanced or metastatic HER2‐positive gastric or gastroesophageal junction adenocarcinoma in patients who had received a prior trastuzumab‐based regimen. Again in 2022, following results from the DESTINY‐Lung02 trial, T‐DXd received FDA approval for the treatment of unresectable or metastatic nonsmall cell lung cancer in adult patients with HER2‐overexpression or HER2 mutations.^71^ Enhertu® comes with an FDA‐boxed warning related to the risk of interstitial lung disease, pneumonitis, and embryo‐fetal toxicity.^64^

Trodelvy® (Sacituzumab govitecan) is the only approved ADC targeting TROP2 as its antigen. It received its first approval in 2020 for treating metastatic triple‐negative breast cancer in adult patients who had undergone at least two prior therapies for metastatic disease.^72^ In 2021, it also gained approval for treating metastatic urothelial cancer. Sacituzumab govitecan comprises a humanized anti‐TROP2 IgG1 antibody linked by a hydrolyzable hydrazone linker to SN‐38, a topoisomerase‐1 inhibitor and the active metabolite of irinotecan. Besides its DNA‐damaging effect within the internalized cell, SN‐38 demonstrates a bystander effect due to its high membrane permeability.^53^, ^72^ In 2023, Sacituzumab govitecan received extended FDA approval for treating patients with hormone‐positive and HER‐2/NEU‐negative metastatic breast cancer with a boxed warning for neutropenia and diarrhea.^53^, ^73^

Tivdak® (Tisotumab vedotin‐tftv [TV]), is a first‐in‐class tissue factor (TF)‐directed ADC approved in 2021 for treating recurrent or metastatic cervical cancer in adult patients. About 10%–20% of patients with early‐stage disease and 70% patients with locally advanced disease experience relapse within 2 years of diagnosis. Only a small fraction of these patients are responsive to curative treatment, necessitating the need for more targeted treatment alternatives.^74^ TV, a humanized IgG1 antibody conjugated to MMAE via a protease‐cleavable linker,^74^ demonstrated clinically meaningful and durable antitumor activity in a Phase 2 clinical study, with target lesions reduced in 79% of treated patients.^75^, ^76^ With more than 50% of patients in the innovaTV 201 and innova TV 204 trials developing ocular related adverse effects, Tivdak® comes with an FDA blackbox warning for ocular toxicity.^64^

ELAHERE™ (mirvetuximab soravtansine‐gynx) is another first‐in‐class ADC approved for treating adult patients with a folate receptor‐α (FRα)‐positive, platinum‐resistant epithelial ovarian, fallopian tube, or primary peritoneal cancer.^77^, ^78^ Approved for patients not responding to platinum‐based chemotherapy who have undergone other types of chemotherapy, it features a chimeric anti‐FRα IgG1 antibody conjugated to a maytansine derivative (DM4) via a cleavable disulfide linker.^78^ Upon endosomal/lysosomal cleavage, DM4 causes cell cycle arrest and apoptosis. DM4 also exhibits a bystander‐killing effect.^53^ ELAHERE™ also comes with an FDA‐boxed warning for ocular toxicity.

## 4 ADCs IN ACTIVE CLINICAL TRIALS

Since the approval of the first ADC in 2000, continued efforts have focused on designing new ADCs with improved efficacy and reduced toxicity. These efforts are evident by the number of ADCs currently in active trials, which represent only a small portion of all ADC research. We conducted a search on clinicaltrials.gov to identify active clinical trials for both approved and newer investigative ADCs. For trials related to approved ADCs, we conducted the search by inputting the drug name in the “Other terms” category for each approved ADC, while checking off “interventional studies,” in active status (“not yet recruiting,” “recruiting,” “enrolling by invitation,” and “active, not recruiting”). For trials related to new ADCs, we conducted the search by using the keywords “antibody drug conjugate OR antibody‐drug conjugate OR ADC OR ADCs OR antibody drug conjugates OR antibody‐drug conjugates” in the “Other terms” category on clinicaltrials.gov, and also checked off “interventional studies,” in active status (“not yet recruiting,” “recruiting,” “enrolling by invitation,” and “active, not recruiting”). All the collected trials were then manually screened to only include trials focusing on IgG‐based ADCs bearing pan‐cytotoxic payloads. Our search identified a total of 551 active clinical trials as of October 2023. Here, we discuss these active trials and highlight new trends emerging from the investigative ADCs in active trials compared with approved products. Tables 2 and 3 present representative active trails for approved ADCs and new investigative ADCs.

**TABLE 2:** Representative active clinical trials for approved ADCs.

	ADC name	mAB	Antigen	Payload	Payload class	Approved indication	No. of active trials	Examples of active trials	
								Indication	ID
Hematological tumors	Brentuximab vedotin	IgG1	CD30	MMAF	Auristatin	Relapsed and/or refractory (R/R) systemic anaplastic large cell lymphoma (2011); R/R primary cutaneous anaplastic large cell lymphoma or CD30+ mycosis fungoides (2017); Classical Hodgkin lymphoma, systemic anaplastic large cell lymphoma, or CD30+ peripheral T‐cell lymphoma (2018)	79	Hodgkin lymphoma	NCT02166463
								T‐cell lymphoma	NCT05442554
								Mycosis fungoides, Sezary Syndrome, Lymphomatoid Papulosis	NCT03587844
	Gemtuzumab ozogamicin	IgG4	CD33	Ozogamicin	Calicheamicin	Relapsed acute myelogenous leukemia (2017)	30	R/R CD33+ acute myeloid leukemia	NCT04070768
								Acute myelogenous leukemia and myelodysplastic syndrome	NCT02221310
								Acute myeloid leukemia	NCT03568994
	Belantamab mafodotin‐blmf	IgG1	BCMA	MMAF	Auristatin	R/R multiple myeloma after at least 4 prior therapies including an anti‐CD38 mAb, a proteasome inhibitor, and an immunomodulatory agent (2020)	14	Recurrent plasma cell myeloma, refractory plasma cell myeloma	NCT05847569
								Multiple myeloma	NCT05064358
								Transplant ineligible newly diagnosed multiple myeloma	NCT05573802
	Loncastuximab tesirine‐lpyl	IgG1	CD19	SG3199	PBD dimer	R/R large B‐cell lymphoma after 2 or more lines of systemic therapy, including diffuse large B‐cell lymphoma (DLBCL) not otherwise specified, DLBCL arising from low‐grade lymphoma, and high‐grade B‐cell lymphoma (2021)	10	Waldenstrom macroglobulinemia	NCT05190705
								R/R B‐cell non‐Hodgkin lymphoma	NCT04970901
								B‐cell lymphoid malignancies	NCT05270057
	Moxetumomab pasudotox	IgG4	CD22	Pseudomonas exotoxin	Pseudotox	Adults with R/R hairy cell leukemia (2018)	1	Hairy cell leukemia	NCT03805932
	Polatuzumab vedotin‐piiq	IgG1	CD79b	MMAE	Auristatin	R/R DLBCL (2019)	10	Richter's transformation	NCT04679012
								R/R mantle cell lymphoma	NCT04659044
								High‐risk diffuse large b‐cell lymphoma	NCT04323956
	Inotuzumab ozogamicin	IgG4	CD22	Ozogamicin	Calicheamicin	R/R CD22‐positive B‐cell precursor acute lymphoblastic leukemia (2017)	26	Acute lymphocytic leukemia	NCT05456698
								B‐cell acute lymphoblastic leukemia	NCT05016947
								Precursor cell lymphoblastic leukemia	NCT03460522
Solid tumors	Ado‐trastuzumab emtansine	IgG1	HER2	DM1	Maytansinoid	HER2+ metastatic breast cancer previously treated with trastuzumab and a taxane (2013); HER2+ early breast cancer after neoadjuvant taxane & trastuzumab‐based treatment (2019)	75	Brain metastases	NCT05323955
								HER2‐positive breast cancer, ER‐negative breast cancer, PR‐negative breast cancer, Node‐negative breast cancer	NCT04675827
								HER2‐positive Salivary Gland Carcinomas	NCT04620187
	Disitamab vedotin	IgG1		MMAE	Auristatin	Patients with locally advanced or metastatic gastric cancer (including gastroesophageal junction adenocarcinoma) who have received at least 2 types of systemic chemotherapy (2021)	23	Her2 overexpressing high‐risk nonmuscle invasive bladder urothelial carcinoma	NCT05495724
								Colorectal Neoplasms	NCT05493683
								Nonsmall cell lung cancer, ERBB2 mutation‐related tumors	NCT05847764
	Fam‐trastuzumab deruxtecan‐nxki	IgG1		DXd	Camptothecin	Unresectable or metastatic HER2+ breast cancer after 2 or more anti‐HER2 regimens (2019) d; locally advanced or metastatic HER2+ gastric or gastroesophageal junction adenocarcinoma after a trastuzumab‐based regimen (2021)	19	Advanced solid tumor	NCT05097599
								HER2+ breast cancer with brain metastasis	NCT05376878
								Locally advanced breast cancer, metastatic breast cancer	NCT05744375
	Tisotumab vedotin‐tftv	IgG1	Tissue factor	MMAE	Auristatin	Recurrent or metastatic cervical cancer with disease progression on or after chemotherapy (2021)	3	Colorectal neoplasms, carcinoma, nonsmall‐cell lung, exocrine pancreatic cancer, carcinoma squamous cell of head and neck	NCT03485209
								Cervical cancer	NCT03786081
								Cervical cancer	NCT04697628
	Sacituzumab govitecan‐hziy	IgG1	TROP2	SN‐38	Camptothecin	Locally advanced or metastatic TNBC after at least two prior therapies (2020); locally advanced or metastatic urothelial cancer after a Pt‐containing chemotherapy and a PD‐1 or PD‐L1 inhibitor (2021)	35	Nonsmall cell lung cancer	NCT05089734
								Metastatic solid tumor	NCT04319198
								Cervical cancer	NCT05838521
	Mirvetuximab soravtansine	Undisclosed	FRα	DM4	Maytansinoid	Ovarian cancer (2022)	13	Endometrial cancer	NCT03835819
								Epithelial ovarian cancer, peritoneal cancer, fallopian tube cancer	NCT05622890
								Platinum‐resistant ovarian cancers	NCT05483933
	Enfortumab vedotin‐ejfv	IgG1	Nectin‐4	MMAE	Auristatin	Locally advanced or metastatic urothelial cancer after a PD‐1 or PD‐L1 inhibitor and a Pt‐containing chemotherapy (2019) or are ineligible for cisplatin‐containing chemotherapy and previously received 1 or more lines of therapy (2021)	15	Metastatic urothelial carcinoma	NCT04963153
								Muscle invasive bladder cancer	NCT04960709
								Nonmuscle invasive bladder cancer	NCT05014139

Abbreviations: ADCs, antibody‐drug conjugates; IgG, Immunoglobulin G; mAB, Monoclonal antibody; MMAE, monomethyl auristatin E; MMAE, monomethyl auristatin E; MMAF, monomethyl auristatin F; TNBC, triple‐negative breast cancer.

**TABLE 3:** Representative active clinical trials for new investigative ADCs.

	ADC name	mAB	Antigen	Payload	Payload class	Linker type	No. of trials	Examples of trials	
								Indication	ID
Hematological tumor	IMGN632	IgG1	CD123	IGN	Monoimine	Cleavable peptide	2	Leukemia	NCT03386513
	ADCT‐301		CD25	SG3199	Tesirine	Cleavable	2	Relapsed hodgkin lymphoma; refractory hodgkin lymphoma	NCT04052997
	SAR3419		CD19	DM4	Maytansinoid	Cleavable	1	Acute lymphocytic leukemia	NCT01440179
	BN301		CD74	Maytansoid	Maytansinoid	Noncleavable	1	Lymphoma	NCT05611853
	STRO‐001		CD74	Maytansinoid	Maytansinoid	Noncleavable	1	Lymphoma	NCT03424603
	INA03	IgG4	CD71	MMAE	auristatin	Undisclosed	1	Leukemia	NCT03957915
	ADCT‐602	IgG2	CD22	SG3249	PBD	Cleavable	1	Leukemia	NCT03698552
	STI‐6129	Unspecified	CD38	Duostatin	Duostatin	Noncleavable (C‐lock chemical linker)	4	Multiple myeloma	NCT05308225
	F0002‐ADC		CD30	DM1	Maytansinoid	Noncleavable (Stable SMCC linker)	1	Refractory or recurrent CD30+ hematologic malignancies	NCT03894150
	CC‐99712		BCMA	Undisclosed	Undisclosed	Noncleavable	1	Multiple myeloma	NCT04036461
	JBH492		CCR7	DM4	Maytansinoid	Cleavable	1	Non‐Hodgkins lymphoma; chronic lymphocytic leukemia	NCT04240704
	IKS03		CD19	Femtogenix's sequence‐selective DNA‐interactive payload molecule	Pyrrolobenzodiazepine	Cleavable	1	Lymphoma	NCT05365659
	CS5001		ROR1	Undisclosed	PBD	Cleavable	1	Advanced lymphoma	NCT05279300
	MRG001		CD20	MMAE	Auristatin	Cleavable (Valine‐citrulline)	1	Relapsed or refractory B‐cell non‐Hodgkin lymphoma	NCT05155839
Solid tumors	BYON3521	IgG1	c‐MET	Duocarmycin	Duocarmycin	Cleavable	1	Solid tumor	NCT05323045
	STRO‐002		Folate receptor alpha (FolRα)	Dibenzocyclooctyne	3‐aminophenyl‐hemiasterlin	Cleavable	2	Ovarian cancer|; fallopian tube cancer; primary peritoneal carcinoma	NCT05200364
	U3‐1402		HER3	Deruxtecan	TOP1i	Cleavable	5	Breast cancer	NCT04610528
	XMT‐1536		NaPi2b	AF‐HPA	Auristatin	Cleavable	3	High grade serous ovarian cancer; fallopian tube cancer; primary peritoneal cancer	NCT05329545
	ARX788		HER2	AS269	Amberstatin/Auristatin	Noncleavable	5	Breast neoplasms	NCT04983121
	MORAb‐202		FolRα	Eribulin	Halichondrin	Cleavable	3	Solid tumor	NCT04300556
	SYD1875		5T4	Duocarmycin	Duocarmycin	Cleavable	1	Solid tumor	NCT04202705
	DS‐8201a		HER2	Deruxtecan	TOP1i	Cleavable	4	Breast tumors	NCT04132960
	CAB‐AXL‐ADC		AXL	MMAE	Auristatin	Cleavable	2	Nonsmall‐cell lung cancer (NSCLC)	NCT04681131
	DS‐7300A		B7‐H3	Deruxtecan	TOP1i	Cleavable	1	Extensive‐stage small‐cell lung cancer	NCT05280470
	IMGC936		ADAM9	DM21	Maytansinoid	Cleavable	1	Advanced solid tumor	NCT04622774
	OBT076		Ly75/CD205	DM4	Maytansinoid	Cleavable	1	Solid tumor	NCT04064359
	CAB‐ROR2‐ADC		ROR2	MMAE	Auristatin	Cleavable	1	NSCLC; triple negative breast cancer; melanoma; head and neck cancer	NCT03504488
	NBE‐002		ROR1	PNU	TOP1i	Noncleavable	1	Advanced solid tumor; triple negative breast cancer	NCT04441099
	DS‐1062a		TROP2	Exatecan	TOP1i	Cleavable	1	Metastatic lung cancer	NCT04940325
	GQ1001		HER2	DM1	Maytansinoid	Undisclosed	2	HER2‐positive breast cancer; HER2‐positive gastric cancer; advanced solid tumor	NCT04450732
	SYD985		HER2	Duocarmycin	Duocarmycin	Cleavable	5	Endometrial cancer	NCT04205630
	HS‐20093		B7‐H3	TOP1i	TOP1i	Cleavable	2	Advanced solid tumor	NCT05276609
	OBI‐999		anti‐globo H	MMAE	Auristatin	Cleavable	1	Locally advanced solid tumor	NCT04084366
	XMT‐1592		NaPi2b	AF‐HPA	Auristatin	Undisclosed	1	Ovarian cancer; NSCLC	NCT04396340
	ABBV‐399		cMET	MMAE	Auristatin	Cleavable	1	Advanced solid tumors cancer	NCT02099058
	ARX517		PSMA	AS269	Amberstatin	Noncleavable	1	Advanced solid tumor; solid neoplasm	NCT04662580
	Dato‐DXd		TROP2	Deruxtecan	TOP1i	Cleavable	6	Carcinoma, nonsmall‐cell lung; triple negative breast cancer	NCT05460273
	BB‐1705		EGFR	Eribulin	Halichondrin	Cleavable	1	Solid tumor	NCT05217693
	MRG002		HER2	MMAE	Auristatin	Cleavable	9	Breast cancer with liver metastases	NCT05263869
	ADC‐1013		CD40	Undisclosed	Undisclosed	Undisclosed	1	Metastatic pancreatic ductal adenocarcinoma	NCT04888312
	ADCT‐301		CD25	SG3199	Tesirine	Cleavable	1	Advanced solid tumors; head and neck cancer squamous cell carcinoma; NSCLC; gastrointestinal cancers; bladder cancer; renal cell carcinoma; melanoma; triple‐negative breast cancer; ovarian cancer; fallopian tube cancer	NCT03621982
	MRG003		EGFR	MMAE	Auristatin	Cleavable (Valine‐citrulline)	7	Advanced or metastatic gastric cancer; advanced or metastatic gastroesophageal junction carcinoma	NCT05188209
	BA3021		Ror2	MMAE	Auristatin	Cleavable	1	Head and neck cancer	NCT05271604
	SGN‐LIV1A		LIV‐1	MMAE	Auristatin	Protease‐Cleavable	1	HER2 positive breast neoplasms; hormone receptor‐positive breast neoplasms; triple negative breast neoplasms	NCT01969643
	MYTX‐011		c‐MET	MMAE	Auristatin	Cleavable	1	NSCLC	NCT05652868
	PYX‐201		Fibronectin extra‐domain B (ED‐B)	Aur0101	Auristatin	Cleavable	1	Solid tumor; advanced solid tumor	NCT05720117
	HuMax‐AXL‐ADC (Enapotamab vedotin)		AXL	MMAE	Auristatin	Protease Cleavable	1	Ovarian cancer; cervical cancer; endometrial cancer; NSCLC; thyroid cancer; melanoma; sarcoma; solid tumors	NCT02988817
	SHR‐A1811		EGFR	SHR9265	TOP1i	Cleavable	5	Breast cancer	NCT05824325
	SGN‐CD228A		CD228	MMAE	Auristatin	Cleavable (Novel glucuronide linker)	1	Cutaneous melanoma; pleural mesothelioma; HER2 negative breast neoplasms; NSCLC; colorectal cancer; pancreatic ductal adenocarcinoma	NCT04042480
	ABT‐414		EGFR	MMAF	Auristatin	Noncleavable	1	Glioblastoma; gliosarcoma	NCT02573324
	ADCT‐901		KAAG1	SG3199	Tesirine	Cleavable	1	Advanced solid tumors	NCT04972981
	BAY 94–9343		MF‐T	DM4	Maytansinoid	Cleavable	1	NSCLC	NCT03455556
	SOT102		Claudin‐18.2	PNU‐159682	TOPi	Noncleavable	1	Gastric cancer; pancreatic cancer; gastro‐esophageal junction cancer	NCT05525286
	BMS‐986148		Mesothelin	Tubulysin	Tubulysin	Cleavable (Valine‐citrulline)	1	Advanced cancer	NCT02341625
	RC48‐ADC	IgG4	HER2	MMAE	Auristatin	Cleavable	21	Upper urinary tract urothelial carcinoma	NCT05912816
	REGN5093‐M114		MET	M114	Maytansinoid	Protease Cleavable	1	Advanced NSCLC	NCT04982224
	ASN004		5T4	Dolaflexin	Auristatin	Noncleavable (Dolaflexin‐drug linker)	1	Breast cancers; |NSCLC; colorectal cancer; ovarian cancer	NCT04410224
	BGB‐A317		PD‐1	Undisclosed	Undisclosed	Undisclosed	1	HER2‐positive or mutated advanced colorectal cancer	NCT05350917
	TR1801‐ADC	IgG2	c‐MET	SG3249	PBD	Cleavable	1	Unspecified adult solid tumor, protocol‐specific	NCT03859752
	SHR‐A1403		c‐MET	Undisclosed	Undisclosed	Noncleavable	1	Advanced solid tumor	NCT03856541
	FDA022	Unspecified	HER2	Undisclosed	Undisclosed	Undisclosed	1	Advanced solid tumors	NCT05564858
	IMGN151		FolRα	DM21	Maytansinoid	Cleavable, peptide	1	Endometrial cancer; ovarian cancer; primary peritoneal carcinoma; fallopian tube cancer	NCT05527184
	RC108		c‐MET	MMAE	Auristatin	Undisclosed	1	Digestive cancer	NCT05628857
	TQB2102		HER2	TOP1i	TOP1i	Cleavable, enzyme	1	Advanced cancer	NCT05735496
	JSKN003		HER2	TOP1i	TOP1i	Cleavable (Dibenzocyclooctyne tetrapeptide linker)	1	Advanced solid tumors; metastatic solid tumors	NCT05494918
	M9140		CEACAM5	DM4	Maytansinoid	Cleavable	1	Colorectal cancer	NCT05464030
	RC118‐ADC		Claudin‐18.2	MMAE	Auristatin	Cleavable	1	Advanced solid tumor	NCT05205850
	SKB264		TROP2	Belotecan	Camptothecin	Cleavable (Sulfonyl pyrimidine‐CL2A‐carbonate)	3	Ovarian epithelial cancer; gastric adenocarcinoma; breast cancer; urothelial carcinoma; NSCLC; small‐cell lung cancer	NCT04152499
	RC88		MSLN	MMAE	Auristatin	Cleavable	1	Solid tumor	NCT04175847
	AZD8205		B7‐H4	TOP1i	TOP1i	Cleavable	1	Breast cancer; cholangiocarcinoma; ovarian cancer; endometrial cancer	NCT05123482
	PF‐06647020		PTK7	Aur0101	Auristatin	Cleavable	1	Cancer; NSCLC	NCT04189614
	W0101		IGF‐R1	Auristatin	Auristatin	Noncleavable	1	Advanced/metastatic solid tumors	NCT03316638
	FDA018‐ADC		TROP2	Undisclosed	Undisclosed	undisclosed	1	Advanced/metastatic solid tumors	NCT05174637
	TORL‐2‐307‐ADC		Claudin‐18.2	MMAE	Auristatin	Undisclosed	1	Advanced solid tumor; gastric cancer; Pancreas cancer; Gastroesophageal Junction Adenocarcinoma	NCT05156866
	STI‐3258		TROP2	Undisclosed	Undisclosed	Undisclosed	1	Solid tumor	NCT05060276
	M1231		EGFR, MUC1	Hemiasterlin	Hemiasterlin	Cleavable	1	Metastatic solid tumors; esophageal cancer; NSCLC	NCT04695847
	YL201		TAA	Undisclosed	Undisclosed	Cleavable (TMALIN)	1	Advanced solid tumor	NCT05434234
	TORL‐1‐23		Claudin 6	MMAE	Auristatin	Cleavable	1	Advanced solid tumor; ovarian cancer; endometrial cancer	NCT05103683
	CPO102		Claudin‐18.2	MMAE	Auristatin	Noncleavable	1	Pancreatic cancer; gastric cancer	NCT05043987
	A166		HER2	Undisclosed	Auristatin	Cleavable	1	Breast cancer	NCT05311397
	A166		HER2	Undisclosed	Auristatin	Cleavable	2	Breast cancer; gastrointestinal cancer; salivary gland cancer; |lung cancer; colo‐rectal cancer; head and neck cancer; bladder cancer; cervical cancer; liver cancer; bile duct cancer; prostate cancer; ovarian carcinoma	NCT03602079
	FOR46		CD46	MMAE	Auristatin	Undisclosed	2	Prostate cancer metastatic	NCT03575819
	IMMU‐132		TROP2	SN‐38	Camptothecin	Undisclosed	1	Prostate cancer	NCT03725761
	SKB315		Claudin‐18.2	TOP1i	TOP1i	Uncleavable	1	Advanced solid tumors	NCT05367635
	ADCT‐601		AXL	SG3199	Tesirine	Cleavable	1	Advanced solid tumors	NCT05389462
	HER3‐DXd		HER3	DXd	TOP1i	Cleavable	3	Metastatic Breast Cancer	NCT02980341
	XB002		TF	MMAE	Auristatin	Cleavable	1	NSCLC; urothelial cancer; ovarian cancer; cervical cancer; pancreatic cancer; prostate cancer; breast cancer	NCT04925284
	BIO‐106		TROP2	Undisclosed	Undisclosed	Undisclosed	1	Advanced solid tumor|	NCT05320588
	MRG004A		TF	MMAE	Auristatin	Cleavable	1	Advanced or metastatic solid tumors	NCT04843709
	CX‐2029		CD71	MMAE	Auristatin	Cleavable	1	Solid tumor, head and neck cancer; NSCLC; diffuse large B‐cell lymphoma; esophageal cancer	NCT03543813
	SKB264		TROP2	Belotecan	Camptothecin	Cleavable (Sulfonyl pyrimidine‐CL2A‐carbonate)	3	NSCLC	NCT05351788
	IBI354		HER2	Undisclosed	Camptothecin	Undisclosed	1	Locally advanced unresectable or metastatic solid tumors	NCT05636215
	TQB2103		Claudin‐18.2	DDDXD	TOP1i	Cleavable	1	ADVANCED malignant neoplasm	NCT05867563
	FZ‐AD004		TROP2	TOP1i	TOP1i	Undisclosed	1	Advanced and metastatic solid tumor	NCT05914545
	DS‐3939a		TA‐MUC1	DxD	TOP1i	Cleavable	1	Advanced/ metastatic solid tumor	NCT05875168
	AMT‐151		FolRα	Undisclosed	Undisclosed	Undisclosed	1	Advanced solid tumor; ovarian cancer; endometrial cancer; lung adenocarcinoma; triple negative breast cancer; pancreatic ductal adenocarcinoma	NCT05498597
	BAT8008		TROP2	Exatecan	TOP1i	Cleavable	1	Advanced solid tumors	NCT05620017
	B003		HER2	DM1	Maytansinoid	Noncleavable (Nonreduceable thioether linkage)	1	HER2‐positive breast cancer	NCT03953833
	STI‐6129		CD38	Duostatin	Duostatin	Noncleavable (C‐lock chemical linker)	1	Advanced solid tumor	NCT05584709
	BAT8009		B7‐H3	Exatecan	TOP1i	Cleavable	1	Locally advanced/metastatic solid tumors	NCT05405621
	OMTX705		MTX5	TAM558	Tubulysin	Cleavable	1	Advanced solid tumor	NCT05547321
	KM501		HER2	Undisclosed	Undisclosed	Undisclosed	1	Advanced solid tumors	NCT05804864
	EBC‐129		CEACAM5	MMAE	Auristatin	Undisclosed	1	Advanced solid tumors	NCT05701527
	BAT8007		Nectin‐4	TOP1i	TOP1i	Cleavable	1	Advanced solid tumors	NCT05879627
	AZD5335		FolRα	TOP1i	TOP1i	Undisclosed	1	Ovarian cancer; lung adenocarcinoma	NCT05797168
	AZD9592		EGFR, c‐MET	TOP1i	TOP1i	Cleavable	1	Advanced solid tumors; carcinoma nonsmall cell lung; head and neck neoplasms	NCT05647122
	MHB088C		B7‐H3	TOP1i	TOP1i	Cleavable	1	Advanced or metastatic solid tumors	NCT05652855
	PRO1184		FolRα	Exatecan	TOP1i	Cleavable	1	Ovarian cancer; primary peritoneal carcinoma; fallopian tube cancer; endometrial cancer; NSCLC; mesothelioma; breast cancer	NCT05579366
	IKS014		HER2	MMAE	Auristatin	Cleavable (Beta‐glucuronide)	1	Breast cancer; gastric cancer; gastroesophageal‐junction cancer	NCT05872295
	HS‐20089		B7‐H4	TOP1i	Undisclosed	Undisclosed	1	Advanced solid tumor	NCT05263479
	9 MW2821		Nectin‐4	MMAE	Auristatin	Undisclosed	2	Advanced malignant solid tumors	NCT05216965
Other diseases	STI‐6129		CD38	Duostatin	Duostatin	Noncleavable (C‐lock chemical linker)	2	Light chain (al) amyloidosis	NCT04316442
								Light chain (AL) amyloidosis	NCT05692908

Abbreviations: ADCs, antibody‐drug conjugates; AF‐HPA, auristatin F‐hydroxypropylamide; DxD, dexrutecan; IgG, Immunoglobulin G; IGN, indolinobenzodiazepine; mAB, Monoclonal antibody; MMAE, monomethyl auristatin E; MMAE, monomethyl auristatin E; MMAF, monomethyl auristatin F; TOP1i, topoisomerase 1 inhibitor.

### 4.1 Scope of disease indications

**FIGURE 2:**
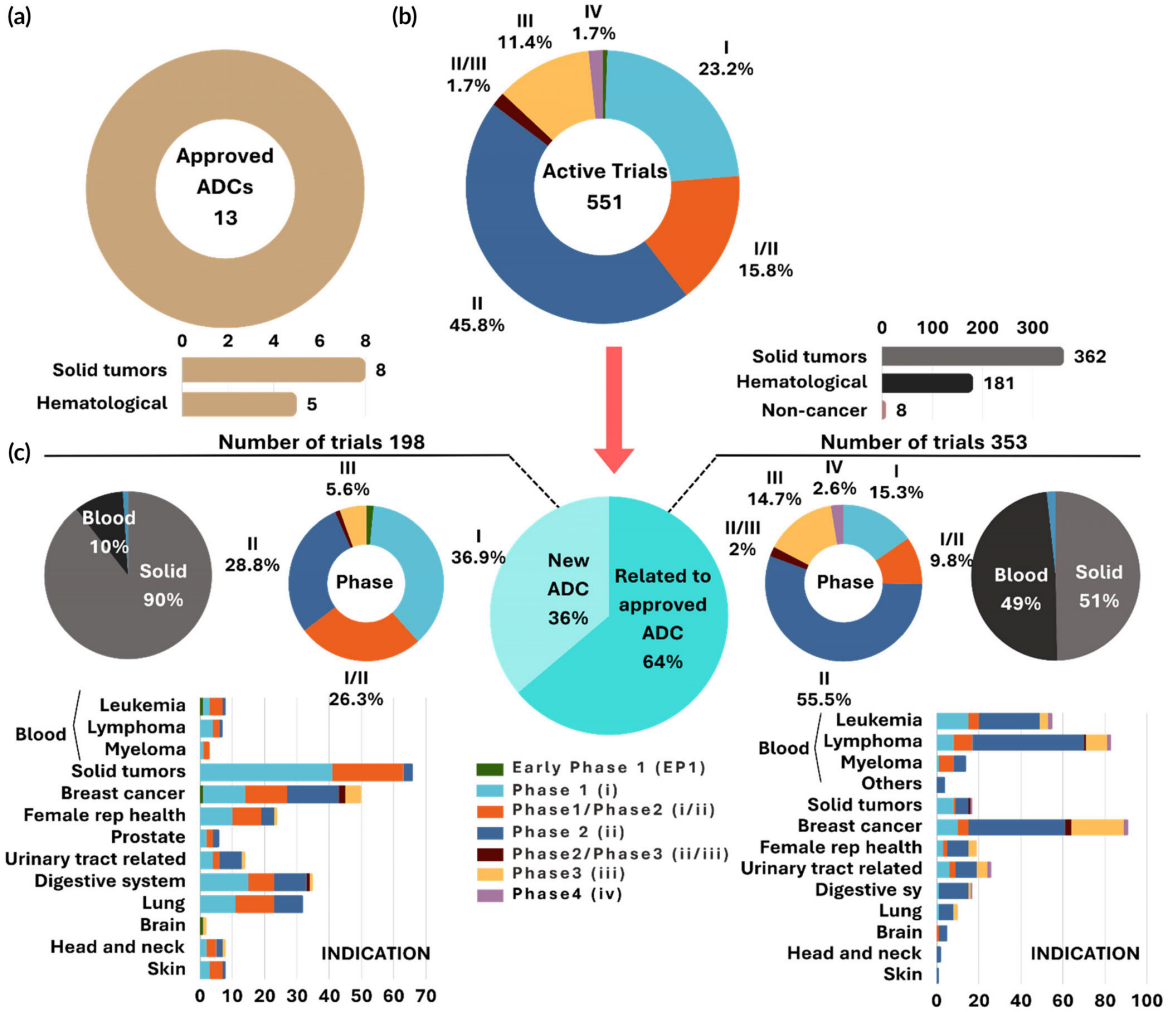
Overview of antibody‐drug conjugates (ADCs) in active clinical trials. (a) Approved ADCs (11 approved by the FDA and 2 approved by other regulatory agencies) in the market showing their scope of disease indications. (b) Phase and disease scope of ADCs in active clinical trials. (c) In‐depth analysis of ADC trials showing the ratio of trials based on new ADC products (left) to trials based on approved ADCs (right) analyzed on phase and indications.

**FIGURE 3:**
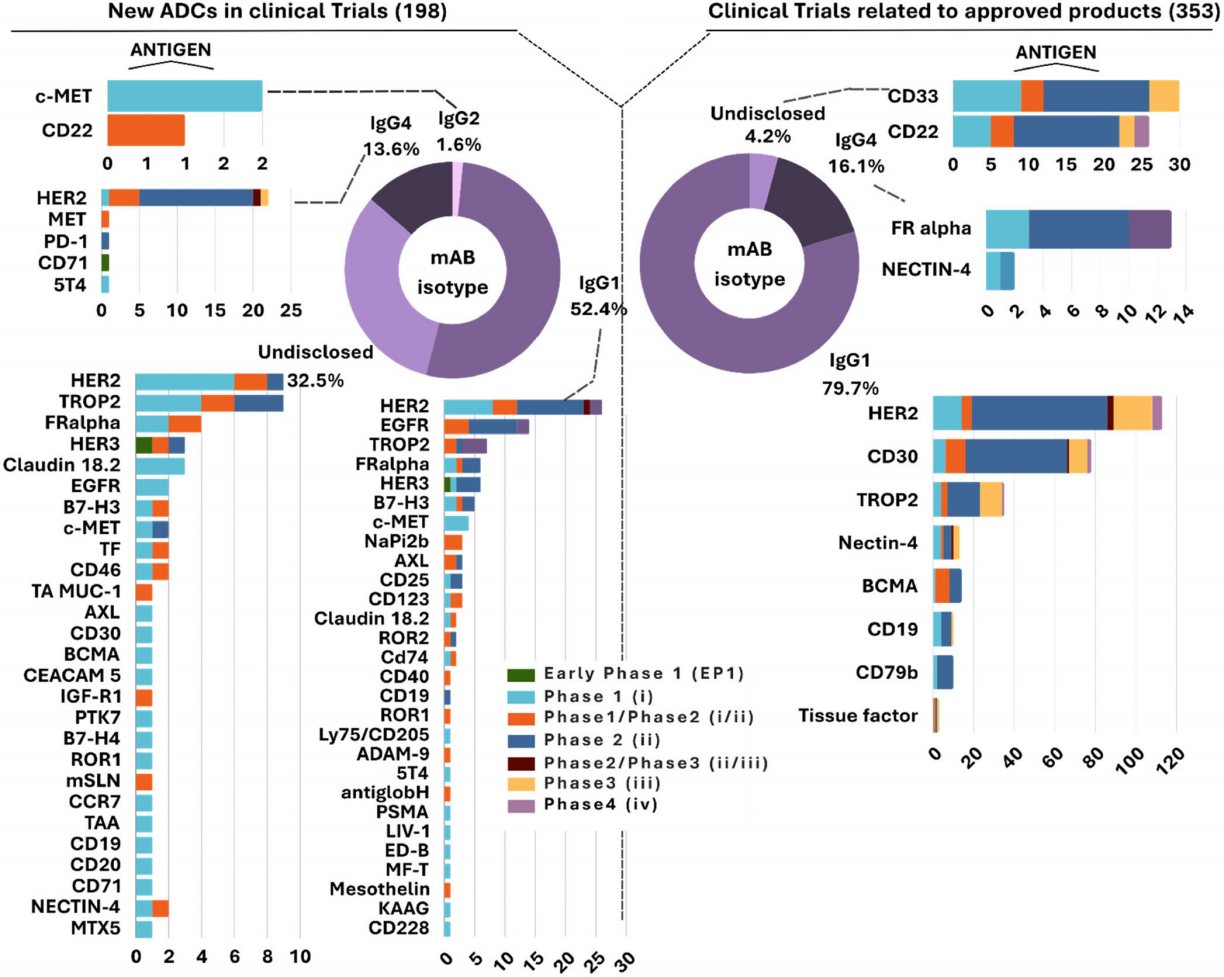
Scope of antibody used in antibody‐drug conjugates (ADCs) under active clinical trials. Comparison between trials for new ADCs (right) and trials for approved ADCs (left) based on mAb isotype. Each isotype is further analyzed (bar chats) to show the range of target‐antigen in different phases of clinical trials.

A large portion of ADC‐focused active trials are geared toward solid tumors, with breast cancer being the most investigated indication, featuring 140 trials. Figure 2 provides an overview of the scope of indications addressed in ADC‐focused active clinical trials. The majority (64%) of these trials aim to expand the clinical application of currently approved products. The ADCs in these trials have the same components as the marketed product but vary in the scope of indications under investigation. Approximately 36% of trials focus on new ADCs that have not yet been approved (Figure 2). When comparing the two groups of interest—trials for new ADCs versus trials related to approved ADCs—a significant shift toward solid tumor applications is noted in the trials for new ADCs, with solid tumor applications representing about 90% of the trials. A detailed breakdown of disease indications for the identified active ADC‐related trials is shown in Figure 2. Another notable observation is the shift in antigen targets, with the second group (trials for new ADCs) showcasing a broader diversity in antigen targets than the first group (Figure 3). There is also a broader diversity in the antibody and drug payload components used in the trials for new ADCs, which all contribute to the wider range of disease indications covered by this group. This diversification is likely driven by the growing understanding of target expression patterns in cancers.

### 4.2 Scope of antibody

The antibody is an essential component of ADCs, as it determines target specificity, thereby enhancing the on‐target cytotoxic effect of the payload.^79^ Moreover, the antibody component influences the plasma concentration, immunogenicity, and immune functions of the ADC and can also contribute to direct or indirect cytotoxic effects.^17^ IgGs are the most used antibodies in ADCs, with the IgG1 subclass being the most prevalent (Figure 3).^79^ About 70% (383 trials) of active clinical trials utilize IgG1, mostly humanized, and likely because of its abundance in serum and high binding affinity to IgG‐binding Fc‐gamma receptors compared with other subclasses, resulting in enhanced antibody‐dependent cytotoxicity and phagocytosis.^17^, ^79^ Following is IgG4, accounting for 15% of active trials. A detailed breakdown of antibody subtypes used in active ADC trials is shown in Figure 3. Additionally, there are three trials involving ADCs made of the IgG2 subclass.

### 4.3 Target antigens

Antigen selection is crucial for the effectiveness and safety of ADCs, as ADCs carry highly potent cytotoxic payloads that require precise delivery to minimize off‐target toxicity. Key considerations in antigen selections include (i) the exclusive or predominant expression of the target on tumor cells for selectivity,^17^, ^79^ (ii) the target antigens' surface expression on tumor cells without their secretion, which could lead to nonspecific drug release,^17^, ^80^ and (iii) the target's ability to trigger cellular internalization of ADCs, crucial for payload delivery.^17^, ^81^

**FIGURE 4:**
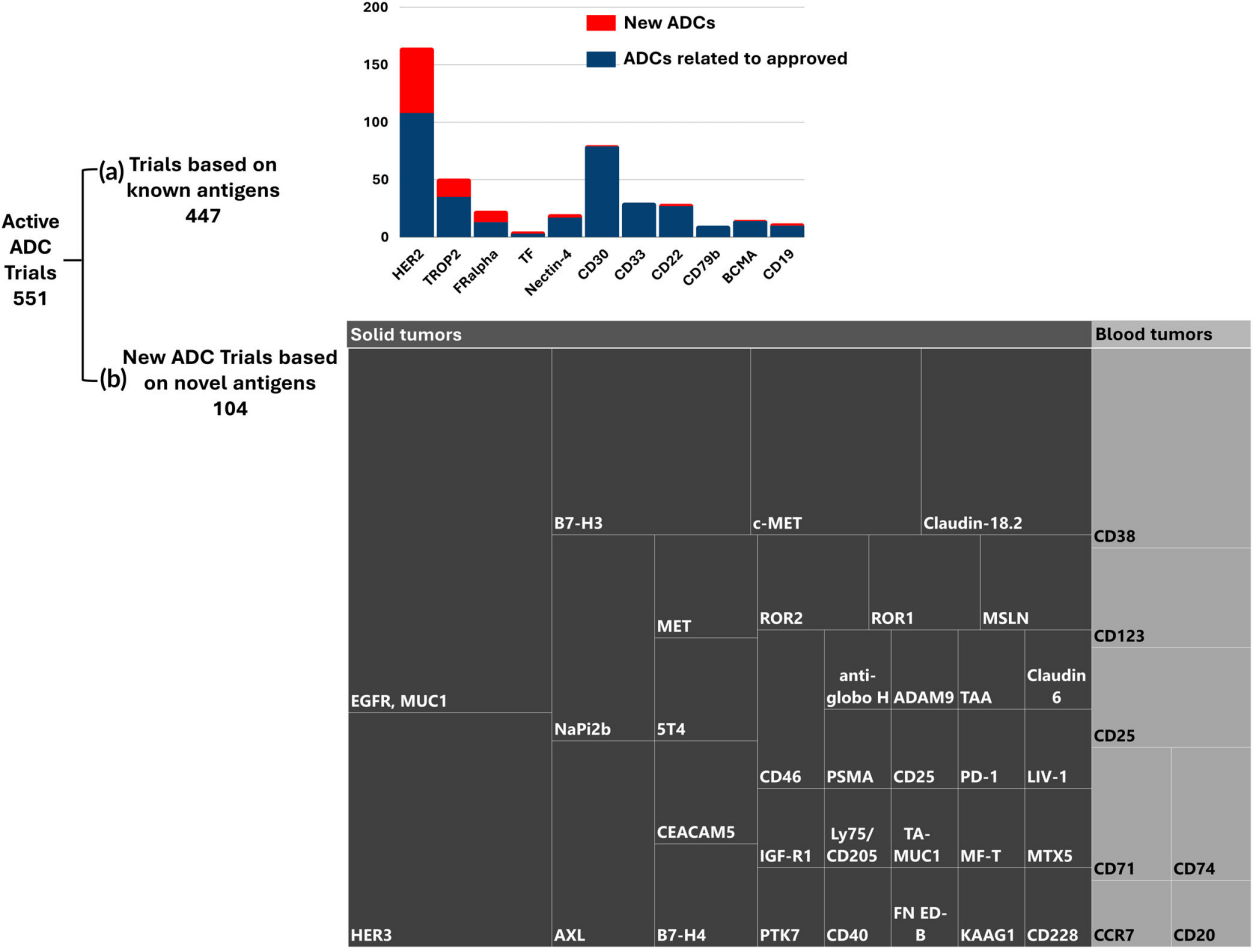
The scope of antigen targets in active antibody‐drug conjugate (ADC)‐focused clinical trials. (a) Bar chart showing the number of trials for new investigative and approved ADCs focusing on known antigens. (b) Tree chart showing the scope of novel antigen targets in trials for new ADC for solid tumors or hematological tumors.

Currently, ADCs approved by the FDA and other regulatory agencies target 11 distinct antigens for hematological malignancies and solid tumors Of the active clinical trials, about 80% (447 trials) focus on these established antigens used in approved products; a detailed breakdown of this is given in Table 2. The leading antigen targets in trials are HER2 (32%), CD30 (14%), and TROP2 (9%). Yet, more novel antigen targets were found in trials for new ADCs, with novel targets accounting for about 20% (104 trials) of total active trials. A detailed breakdown of these novel targets used in active ADC trials is shown in Figure 4. The pursuit of novel targets is a major driver for the extension of ADCs to solid tumors, as the use of these novel targets could potentially reduce ADC's toxicity to normal tissues. While most of these antigens are tumor‐associated rather than tumor‐specific, there is also an extension from typical tumor cell antigens to antigens found in the tumor microenvironment and neovasculature.

#### 4.3.1 Novel targets in active clinical trials—Hematological cancer antigens

Hematological cancers are considered more accessible than solid tumors. This explains why most antigens used in ADCs for treating hematological cancers often pertain to both neoplastic and non‐neoplastic cells, given ADC's direct access to diseased cells. Additionally, the absence of these targets on hematopoietic stem cells and nonhematopoietic tissues allows for the continuous replenishment of blood cells and reduces cytotoxicity, respectively. Classic antigens used in approved ADC formulations for hematological cancers include CD19, CD22, CD30, CD33, CD79b, and BCMA, with detailed reviews available elsewhere.^53^, ^82^ A significant portion of ADC‐focused active clinical trials (32%) targeting these antigens aims to expand the indications of approved ADCs or enhance their efficacy through combinations with other chemotherapeutics (e.g., doxorubicin, cyclophosphamide, gemcitabine) or immunotherapy (e.g., pembrolizumab, nivolumab, rituximab). Currently, there are 18 new ADC trials (3% of total active trials) focusing on hematological tumors, with 14 targeting novel antigens. Details about the scope of these antigens in clinical trials can be found in Table 3. These antigens include CD74, CD20, CCR7, and CD25 for lymphomas; CD71, CD123, CD25, and CD38 for leukemias; and CD38 for myeloma and light chain amyloidosis. These antigens are broadly expressed in immune cells (e.g., B cells, T cells, NK cells, dendritic cells, monocytes, macrophages), erythroid lineage cells, and other tissues as well.

#### 4.3.2 Novel targets in active clinical trials—Solid tumor antigens

Unlike hematological cancer antigens, solid tumor antigens are not lineage‐specific and are mostly tumor‐associated. This means these antigens are mainly overexpressed in tumor cells but may also be expressed at lower levels in healthy cells, raising concerns about off‐target toxicity and reduced intratumoral drug delivery.^82^ Therefore, identifying targets with limited expression in healthy tissues is crucial to improve the therapeutic effectiveness of ADCs. Classical antigens targeted by FDA‐approved ADCs for solid tumor include HER2, TROP2, TF, nectin‐4, and FRα. Currently, 32 novel solid tumor antigens are being investigated in clinical trials for new investigative ADCs, such as B7 family proteins, EGFR, HER3, mesenchymal–epithelial transition factor (c‐MET), AXL, Claudin‐18.2, and NaPi2b accounting for 15% of active trials. Further details about these new antigens and related trials are shown in Figure 4 and Table 4.

**TABLE 4:** Representative active ADC trials involving novel antigens (more than four trials) for solid tumors.

Antigen	ADC name	Condition	Payload	Phase	ID
B7‐H3	DS‐7300A	Extensive‐stage small‐cell lung cancer	Deruxtecan	2	NCT05280470
	MGC018	Cancer: solid tumors	Duocarmycin	1/2 (in combination with retifanlimab)	NCT03729596
				1 (in combination with Lorigerlimab)	NCT05293496
	HS‐20093	Advanced solid tumor	TOP1i	1	NCT05276609
	MHB088C	Advanced or metastatic solid tumors	TOP1i	1/2	NCT05652855
	BAT8009	Locally advanced/metastatic solid tumors	TOP1i	1	NCT05405621
	HS‐20093	Osteosarcoma/sarcoma	TOP1i	2	NCT05830123
EGFR	M1231	Metastatic solid tumors; esophageal cancer; nonsmall cell lung cancer (NSCLC)	Hemiasterlin	1	NCT04695847
	BB‐1705	Solid tumor	Eribulin	1/2	NCT05217693
	MRG003	Advanced or metastatic gastric cancer; advanced or metastatic gastroesophageal junction carcinoma	MMAE	2	NCT05188209
		Recurrent or metastatic nasopharyngeal carcinoma		2	NCT05126719
		Recurrent or metastatic squamous cell carcinoma of head and neck		2	NCT04868162
		Advanced or metastatic biliary tract cancer		2	NCT04838964
		Carcinoma, nonsmall‐cell lung		2	NCT04838548
		Advanced solid tumors		1/2	NCT05688605
		Squamous cell carcinoma of the head and neck		3	NCT05751512
	AZD9592	advanced solid tumors; carcinoma nonsmall cell lung; head and neck neoplasms	TOP1i	1	NCT05647122
	SHR‐A1811	Breast cancer	SHR9265	1/2	NCT05824325
		Triple‐negative breast cancer (TNBC)		2	NCT05749588
		HER2 low breast carcinoma		2	NCT05911958
		Breast neoplasm; breast cancer; hormone receptor positive tumor|HER2‐negative breast cancer; advanced breast cancer		2	NCT05594095
		Breast neoplasm; breast cancer; breast tumors; TNBC; HER2‐positive breast cancer; HER2‐negative breast cancer; hormone receptor positive tumor; hormone receptor negative tumor; early‐stage breast cancer; locally advanced breast cancer		1/2	NCT05582499
	ABT‐414	Glioblastoma; gliosarcoma	MMAF	3	NCT02573324
HER3	U3‐1402	Breast cancer	Deruxtecan	Early Phase 1	NCT04610528
		Metastatic breast cancer		2	NCT04965766
		Metastatic breast cancer; locally advanced breast cancer		2	NCT04699630
		Metastatic colorectal cancer		2	NCT04479436
		NSCLC		1	NCT03260491
	HER3‐DXd	Metastatic breast cancer	TOP1i	1	NCT02980341
		Metastatic breast cancer; advanced nonsmall cell squamous lung cancer; solid tumor		2	NCT05865990
		Brain metastases		Early Phase 1	NCT05620914
c‐MET	BYON3521	Solid tumor	duocarmycin	1	NCT05323045
	RC108	Solid tumor	Undisclosed Microtubule	1	NCT04617314
		Digestive cancer	Inhibitor	2	NCT05628857
	TR1801‐ADC	Unspecified adult solid tumor, protocol‐specific	SG3249	1	NCT03859752
	BYON3521	Solid Tumor	duocarmycin	1	NCT05323045
	MYTX‐011	NSCLC; NSCLC Stage IV|NSCLC Stage IIIB; NSCLC; advanced nonsmall cell squamous lung cancer; advanced NSCLC; advanced nonsmall cell nonsquamous lung cancer	MMAE	1	NCT05652868
	SHR‐A1403	Advanced solid tumor	Undisclosed Microtubule Inhibitor	1	NCT03856541
AXL	CAB‐AXL‐ADC	Nonsmall‐cell lung cancer	MMAE	2	NCT04681131
		Solid tumor; NSCLC; melanoma; sarcoma; sarcoma, ewing; osteosarcoma; leiomyosarcoma; synovial sarcoma; liposarcoma; soft tissue sarcoma; bone sarcoma; refractory sarcoma		1/2	NCT03425279
	ADCT‐601	Advanced solid tumors	SG3199	1	NCT05389462
	HuMax‐AXL‐ADC (Enapotamab vedotin)	Ovarian cancer; cervical cancer; endometrial cancer; NSCLC|thyroid cancer; melanoma; sarcoma; solid tumors	MMAE	1/2	NCT02988817
Claudin 18.2	TORL‐2‐307‐ADC	Advanced solid tumor; gastric cancer; pancreas cancer; gastroesophageal junction adenocarcinoma	MMAE	1	NCT05156866
	RC118‐ADC	Advanced solid tumor	MMAE	1/2	NCT05205850
	SKB315	Advanced solid tumors	TOP1i	1	NCT05367635
	TQB210	Advanced malignant neoplasm	DDDXD	1	NCT05867563
	SOT102	Gastric cancer; pancreatic cancer; gastro‐esophageal junction cancer	PNU‐159682	1/2	NCT05525286
NaPi2b	XMT‐1536	High grade serous ovarian cancer; fallopian tube cancer; primary peritoneal cancer	AF‐HPA	3	NCT05329545
		Platinum‐sensitive ovarian cancer (UPGRADE‐A)		1/2	NCT04907968
		Platinum‐resistant ovarian cancer; NSCLC metastatic		1/2	NCT03319628
	XMT‐1592	Ovarian cancer; NSCLC	Undisclosed	1/2	NCT04396340

Abbreviations: ADC, antibody‐drug conjugates; IgG, Immunoglobulin G; MMAE, monomethyl auristatin E; MMAF, monomethyl auristatin F.

### 4.4 Diversity of payload and linker in active clinical trials

Due to their specificity for tumor cells or tissues, ADCs can minimize the off‐target effects associated with the parent chemotherapeutic drugs.^12^, ^83^ The currently approved ADCs utilize DNA‐damaging and microtubule‐inhibiting payloads, such as auristatins, maytansinoid, camptothecin, and calicheamicin, effective at sub‐nanomolar concentrations.^82^ These payloads are not suitable for systemic administration alone due to their high cytotoxicity. ADCs present a valuable tool for repurposing small‐molecule drugs previously limited by off‐target toxicity.^12^

**FIGURE 5:**
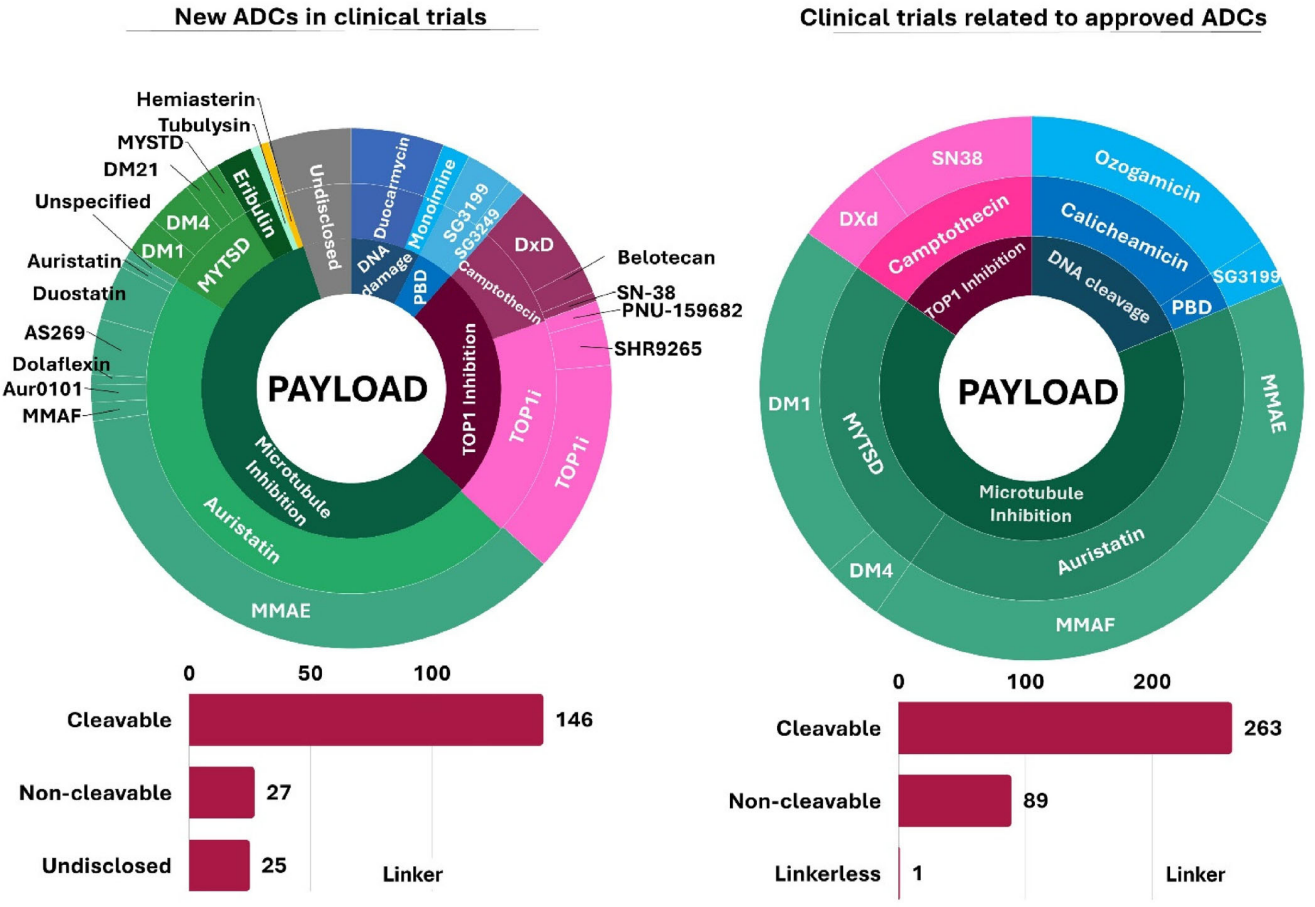
Payload and linker diversity in antibody‐drug conjugate (ADC)‐focused active clinical trials. Pie‐charts showing the drug action (tier 1), class (tier 2), and payload (tier 3) in trials for new ADCs (left) and trials for approved ADCs (right).

The ongoing expansion of indications for ADCs in active clinical trials also reflects the inclusion of new payloads into investigative ADCs. Of the 200 active clinical trials for new ADCs, about a quarter involve new payloads. Figure 5 provides a breakdown of the payloads in current ADC trials, with microtubule inhibitors constituting 51% of these payloads. Auristatins, which inhibit tubulin polymerization, dominate with 81 trials, possibly attributed to their favorable biochemical properties.^82^ Topoisomerase inhibitors, which cause DNA damage through DNA intercalation, represent the second major payload class in ADC trials, totaling 44 trials. Some examples of the new payloads being explored in investigative ADCs include the DNA alkylating clas—duocarmycin (10 trials), PBD dimers and pyridinobenzodiazepines (7 trials), and monoamine indolinobenzodiazepines (7 trials). These molecules are known as highly potent antitumor agents.^84^ Detailed chemical properties of these payloads can be found in recent reviews published elsewhere.^28^

The linker in an ADC significantly impacts the ADC's safety and efficacy. In ADC design, an ideal linker should be stable enough to prevent premature drug release while being sensitive to enable site‐specific drug release.^84^ ADCs are largely designed with either cleavable or noncleavable linkers. The cleavable linkers are responsive to pH changes, glutathione/disulfide isomerase, or proteases present in the TME, while noncleavable linkers rely on the endosomal/lysosomal degradation of the ADCs for drug release.^53^, ^84^ Reflecting the preference in approved ADC products, cleavable linkers are favored in new investigative ADCs in current clinical trials (Figure 5). Examples of such cleavable linkers include glutathione‐sensitive disulfide linkers, protease‐sensitive linkers (phenylalanine‐lysine, valine‐citrulline), and acid‐sensitive hydrazone linkers.

## 5 CHALLENGES AND OUTLOOK FOR CLINICAL TRANSLATION OF ADCs

The principle behind the efficacy of ADCs is straightforward. However, the development of an effective ADC is challenging. To date, improvements such as the use of humanized antibodies, highly potent payloads, and the development of highly stable linkers have driven the development of more effective ADCs with many promising candidates in clinical trials.^20^

A pressing challenge in the development of ADCs is optimizing drug loading. This includes determining the optimal DAR and achieving a homogenous drug conjugation. The DAR is an important property that influences the PK, stability, and efficacy of ADCs.^21^ Different studies have shown the need to link a certain number of drug units to each mAb to optimize its effectiveness. However, a higher DAR does not necessarily imply better efficacy. While ADCs conjugated with a high number of payloads demonstrate increased in vitro potency, several in vivo studies have revealed a negative correlation between high DAR and toxicity and aggregation.^85^, ^86^ An ADC with a DAR of 4 showed equivalent in vivo antitumor activity to that with a DAR of 8, and a further reduction to a DAR of 2 improved in vivo activity.^21^, ^87^, ^88^ An average DAR of four is often recommended, as higher DAR values increase plasma clearance and antibody aggregation, reducing the therapeutic index of ADCs.^87^, ^89^ However, contrary to this general recommendation, a good number of new ADCs have a DAR higher than 4 and are showing promising results in clinical trials. For example, XMT‐1536, a dolaflexin‐based ADC targeting SLC34A2/NaPi2b in solid tumors, has a DAR of up to 15. Although this contradicts the typical DAR recommendations, the payload is a prodrug of auristatin F that exhibits notable bystander cytotoxicity, increasing its antitumor efficacy. Once metabolized intratumorally, auristatin F becomes impermeable to cell membranes, further reducing the systemic drug exposure and improving the overall tolerability.^90^ Thus, this prodrug approach holds potential in improving ADC efficacy, as its overall PK profile is comparable to those of other clinically investigated ADCs with lower DARs.

Early approved ADCs are based on random/heterogeneous conjugation of the payload to the antibody. This approach has been shown to negatively impact the therapeutic index of ADCs.^89^, ^91^ Heterogenicity could translate to each ADC containing an amount of material above the nominal DAR^86^ and/or ADCs with both unconjugated and overloaded antibodies.^89^ Hence, the current research focus is on achieving homogenous ADCs that have the same site of drug attachment between individual mAbs. This goal is technologically challenging and depends on the method of conjugation of the linker to the mAb. The most common method of conjugation is via the lysine side‐chain amines or cysteine sulfhydryl groups.^87^, ^89^ This results in a mixture of species (>100 species) with different DARs, linked at different sites, and each species displaying distinct in vivo PK and efficacy patterns.^87^, ^91^ Purification can be used to eliminate species with different DARs, but this still leaves a heterogeneous mixture with payloads attached at different sites, resulting in batch‐to‐batch variations in ADC production.^91^

In addition, the site of payload attachment and the coupling technique also influence the PK and stability of ADCs.^85^, ^89^, ^92^ Coupling of payloads can induce both physical and chemical instability. For example, in cysteine conjugation, there is a reduction of the interchain disulfide bond on the mAb's free cysteine residue. These cysteines play an important role in maintaining the structure of the antibody, and such disturbance increases the risk of instability.^93^ To overcome these challenges, an emerging approach is to use a site‐specific attachment strategy. This approach involves conjugating payloads to sites that individually minimize the density and solvent accessibility to the hydrophobic payloads.^92^, ^94^ It has been shown that while ADCs with a DAR of 8, only achieved minor tumor inhibition in vivo, using a site‐specific ADC, still with a DAR of 8, resulted in superior tumor inhibition, showing that location matters.^85^ This necessitates investigating mAbs to find specific sites that display plasma exposure equivalent to the unconjugated antibody, thereby improving the therapeutic efficacy of ADCs. Alternatively, another emerging strategy is to adopt other methods of conjugation.^91^, ^95^ A few of the new ADCs in clinical trials adopt novel conjugation methods. SKB264, a TROP‐2 targeting ADC, utilizes a novel coupling strategy that permits the conjugation of seven to eight payloads on the reduced interchain disulfide bonds via a covalent sulfonyl pyrimidine‐CL2A‐carbonate linker. This strategy improves the stability of the ADC and increases its plasma half‐life to up to 57 h in mice. When compared with TRODELVY (an approved TROP‐2 targeting ADC), SKB264 at the same dose demonstrates improved antitumor efficacy and reduced adverse effects.^96^ STI‐6129, another new ADC with six ongoing clinical trials, utilizes this disulfide re‐bridging approach to achieve site‐specific conjugation of five duostatin molecules to an anti‐CD38 mAb. Using this strategy, STI‐6129 shows an internalization rate comparable to that of the unconjugated antibody.^97^ Other strategies to improve conjugation include the introduction of an additional cysteine group at strategic points on the mAbs to preserve the innate cysteine. ADCT‐602 and IMGN632, which contain a cysteine‐engineered anti‐CD22 mAB^98^ and anti‐CD123 mAb,^99^, ^100^ respectively, are notable examples for this strategy. This modification retains antigen binding and specificity and yields homogeneous conjugates.^89^, ^101^ Choosing the right site can thus improve drug loading and reduce clearance.^92^ Other studies have suggested that lysine conjugation could be more beneficial than site‐specific cysteine conjugation,^102^ highlighting the need for a case‐by‐case optimization of conjugation methods in ADC development. There are a few notable ADCs with other site‐specific conjugation strategies in clinical trials. ARX‐788, an amberstatin‐bearing anti‐HER2 ADC with a DAR of 1.4, utilizes a noncleavable linker based on a non‐natural amino acid technology. This results in a homogeneous ADC with high serum stability, outperforming T‐DM1 in preclinical studies.^103^ Likewise, ADCT‐601 utilizes an *N*‐glycosylation site to achieve site‐specific conjugation of SG3199 to an anti‐AXL mAb via a cleavable linker.^104^, ^105^

Another notable challenge affecting the development and clinical application of ADCs is the development of resistance. The advantage of having multiple mechanisms of action also sets ADCs up for resistance, as it can occur at any of these steps.^106^ Resistance to ADCs can be antigen‐related, payload‐related, or tumor‐cell‐related.

Antigen‐related resistance could be due to the reduced expression of antigen or truncated forms of antigen ectodomain, leading to reduced binding of antibodies to the cell surface.^106^, ^107^, ^108^ It has been shown that months of treatment with anti‐HER 2 trastuzumab‐maytansinoid ADC (TM‐ADC) resulted in 16‐fold resistance to TM‐ADC and cross‐resistance to other trastuzumab ADCs, partly due to decreased HER2 expression.^109^ A similar trend in the downregulation of antigen (CD30) has also been reported for resistance to Brentuximab Vedotin.^110^ Tumor heterogenicity^111^, ^112^ and genomic alterations^113^ of antigens can also lead to varied expression of antigens in different parts of the tumor, contributing to treatment failures. Such resistance is typically overcome by switching to other ADCs or standard‐of‐care chemotherapeutics.^109^, ^114^

Payload‐related resistance is mostly due to the overexpression of drug efflux pumps on the tumor cells. Efflux pumps, such as MDR1, also known as permeability glycoprotein1, are responsible for the development of resistance to many small‐molecule drugs and likewise ADCs.^106^, ^109^, ^110^ The overexpression of ABC transporters has been reported to be responsible for resistance to T‐Dxd, T‐DM1, gemtuzumab ozogamicin, and an anti‐CD33‐calicheamicin ADC.^107^, ^115^ Strategies to overcome such resistance include the diversification of payloads, which account for the number of new payloads under ADC trials (Figure 4).^108^ Replacing the tubulin inhibitor DM1 with a topoisomerase inhibitor was reported to effectively overcome T‐DM1 resistance,^115^ and switching from auristatin‐based ADCs to anthracycline‐based ADCs also showed a similar effect.^116^ Other strategies include optimizing DAR and conjugation techniques and developing more hydrophilic ADCs, as MDR1 has a higher preference for hydrophobic compounds.^117^, ^118^

The genetic instability of cancer cells enables them to continually develop mechanisms to evade treatment. Tumor‐cell‐related resistance mechanisms involve changes in trafficking pathways, lysosomal dysfunction, and alterations in apoptotic signaling pathways. Effective internalization of ADCs is central to its mechanism of action (Figure 1) and predominantly occurs via CME.^117^ The use of alternative routes like caveolar‐mediated endocytosis could result in the accumulation of ADCs in caveolin‐1 (CAV1)‐coated vesicles, reduced lysosomal colocalization, and overall reduced efficacy.^117^, ^119^ It was reported that while HER2 expression in some T‐DM1‐resistant cell lines remained normal, intracellular traffic, lysosomal pH, and proteolytic activity were abnormal. Increased lysosomal pH and deranged protease activity result in the accumulation of intact ADC within the cell.^120^ This is particularly noted in ADCs with linkers that require complete proteolysis of the antibody to release payload. Hence, switching to using linkers that only require one proteolytic event could help overcome this mechanism of resistance.^117^ Other strategies involve the use of ADCs with alternative cleavage mechanisms,^121^, ^122^ the use of nanoparticles and other drugs to stabilize lysosomal pH,^123^ and more recently, the use of dual‐drug ADCs and bispecific ADCs. Leveraging insights from these resistance mechanisms, some new ADCs in clinical trials feature new designs to improve their efficacy. Notably, SYD985 and DS‐8201a, ADCs based on trastuzumab, show promise in overcoming T‐DM1 drug resistance in HER2‐positive breast cancers. These second‐generation ADCs employ more potent cytotoxic payloads and have shifted from covalent to cleavable linkers. SYD985, currently in 5 active trials, utilizes a duocarmycin‐derived payload, whose cytotoxicity is not cell cycle dependent, conjugated using a valine‐citrulline linker. Unlike T‐DM1, which requires the complete degradation of the antibody for payload release, SYD985's payload is released through cathepsin‐mediated cleavage of its linker.^124^, ^125^ As another example, DS‐8201a contains a topoisomerase‐inhibiting DXd payload conjugated via a stable tert‐butoxycarbonyl‐glycyl‐glycyl‐phenylalanyl‐glycine linker to trastuzumab. DS‐8201a can circumvent T‐DM1 resistance as DXd is resistant to the effect of p‐glycoproteins, and the stable linker allows for the conjugation of up to eight drug molecules, enhancing its cytotoxicity.^126^ Furthermore, both ADCs exhibit significant bystander cytotoxicity due to the high membrane permeability of the payloads.^126^

Bispecific biparatopic ADCs are an emerging strategy to overcome tumor‐cell‐related ADC resistance. These ADCs are designed to have antibodies with different nanobodies that can simultaneously recognize either different targets or the same targets but different and nonoverlapping epitopes. This helps to improve internalization, lysosomal trafficking, and subsequent degradation of the ADC. A notable example is M1231, a first‐in‐class bispecific ADC targeting MUC‐1 and EGFR, demonstrating superior efficiency in cell internalization and lysosomal trafficking compared with monospecific mAbs.^127^, ^128^ By recognizing different epitopes, this strategy is useful in overcoming resistance due to antigen downregulation.^129^ Furthermore, combining the specificity of two antibodies can also improve ADC potency by blocking targets central to disease progression and/or initiating ADCC or CDC.^130^ It was reported that engaging the overexpressed MET in lung cancers with a biparatopic ADC, METxMET‐M114, provided more benefits than merely blocking the MET function and had the potential to overcome acquired resistance to MET‐selective tyrosine kinase inhibitors.^131^ Numerous other studies have demonstrated the potential of bispecific ADCs, suggesting that this could indeed be a future direction for ADC development.^130^, ^132^, ^133^, ^134^, ^135^ However, it is worth noting that the functioning of biparatopic antibody requires a minimal number of antigen expressions, thus limiting its function below that threshold.^129^

Another emerging strategy to address the problem of ADC resistance incorporates the concept of polypharmacy, but without the complexity of multiple drug regimens. This involves the use of dual‐drug ADCs. These ADCs commonly consist of payloads with different physiochemical properties or mechanisms of action attached to the mAb via the same linker.^136^ By coupling MMAE and MMAF, Levengood et al.^137^ reported a 3:5 cure rate, compared with a 1:5 cure rate achieved with only MMAF. Similarly, another study designed an MMAE and MMAF dual ADC using a click chemistry‐based linker, which allowed for effective control of DAR and more effective tumor killing.^138^ Similarly, it was demonstrated that a dual ADC bearing MMAE and a PBD dimer could achieve tumor killing via different mechanisms.^139^ ADCs designed in this manner have been reported to have increased efficacy compared with the single‐drug formulations and represent a future direction in ADC development.^136^

## 6 CONCLUSION

The surge in the clinical adoption of ADCs since their initial approval is attributed to their superior therapeutic effectiveness over traditional cytotoxic therapies. ADCs have emerged as the primary treatment option for certain blood and solid tumors that are unresponsive to conventional chemotherapy, underscoring their potential in targeting cancers with identifiable markers. Currently, more than 500 clinical trials are exploring numerous new ADCs, suggesting an anticipated increase in ADC approvals across a broader range of indications in the forthcoming years. Insights gained from the clinical use of existing ADCs have spurred the creation of next‐generation ADCs, which promise enhanced efficacy and fewer adverse effects. Innovations including the discovery of novel targets, the refinement of conjugation techniques, optimization of the DAR and the diversification of cytotoxic agents are poised to improve the PK and safety profiles of ADCs significantly. Despite facing challenges such as drug resistance and tumor heterogeneity, ongoing advancements in ADC technology offer optimism for overcoming these obstacles.

## AUTHOR CONTRIBUTIONS

**Edidiong Udofa:** Data curation; validation; writing–original draft; writing–review and editing. **Disha Sankholkar:** Data curation; formal analysis; methodology; writing–original draft; writing–review and editing. **Samir Mitragotri:** Conceptualization; supervision; writing–review and editing. **Zongmin Zhao:** Conceptualization; supervision; writing–review and editing.

## CONFLICT OF INTEREST STATEMENT

SM is a shareholder of Aarvik Therapeutics.

### PEER REVIEW

The peer review history for this article is available at https://www.webofscience.com/api/gateway/wos/peer-review/10.1002/btm2.10677.

## Data Availability

All data are available in the main article.

## References

[1] Padma VV . An overview of targeted cancer therapy. Biomedicine (Taipei). 2015;5(4):19. 26613930 10.7603/s40681-015-0019-4 PMC4662664

[2] Debela DT , Muzazu SG , Heraro KD , et al. New approaches and procedures for cancer treatment: current perspectives. SAGE Open Med. 2021;9:20503121211034366. 34408877 10.1177/20503121211034366 PMC8366192

[3] Zugazagoitia J , Guedes C , Ponce S , Ferrer I , Molina‐Pinelo S , Paz‐Ares L . Current challenges in cancer treatment. Clin Ther. 2016;38(7):1551‐1566. 27158009 10.1016/j.clinthera.2016.03.026

[4] Hou J , He Z , Liu T , et al. Evolution of molecular targeted cancer therapy: mechanisms of drug resistance and novel opportunities identified by CRISPR‐Cas9 screening. Front Oncol. 2022;12:755053. 35372044 10.3389/fonc.2022.755053 PMC8970599

[5] Keefe DMK , Bateman EH . Potential successes and challenges of targeted cancer therapies. J Natl Cancer Inst Monogr. 2019;2019(53):lgz008. 31425592 10.1093/jncimonographs/lgz008

[6] Gerber DE . Targeted therapies: a new generation of cancer treatments. Am Fam Physician. 2008;77(3):311‐319. 18297955

[7] Scott AM , Wolchok JD , Old LJ . Antibody therapy of cancer. Nat Rev Cancer. 2012;12(4):278‐287. 22437872 10.1038/nrc3236

[8] Parakh S , King D , Gan HK , Scott AM . Current development of monoclonal antibodies in cancer therapy. In: Theobald M , ed. Current Immunotherapeutic Strategies in Cancer. Springer International Publishing; 2020:1‐70. 10.1007/978-3-030-23765-3_1 31473848

[9] Zahavi D , Weiner L . Monoclonal antibodies in cancer therapy. Antibodies. 2020;9(3):34. 32698317 10.3390/antib9030034 PMC7551545

[10] Shuel SL . Targeted cancer therapies: clinical pearls for primary care. Can Fam Physician. 2022;68(7):515‐518. 35831091 10.46747/cfp.6807515 PMC9842142

[11] Strebhardt K , Ullrich A . Paul Ehrlich's magic bullet concept: 100 years of progress. Nat Rev Cancer. 2008;8(6):473‐480. 18469827 10.1038/nrc2394

[12] Dumontet C , Reichert JM , Senter PD , Lambert JM , Beck A . Antibody–drug conjugates come of age in oncology. Nat Rev Drug Discov. 2023;22(8):641‐661. 37308581 10.1038/s41573-023-00709-2

[13] Fuentes‐Antrás J , Genta S , Vijenthira A , Siu LL . Antibody–drug conjugates: in search of partners of choice. Trends Cancer. 2023;9(4):339‐354. 36746689 10.1016/j.trecan.2023.01.003

[14] Diamantis N , Banerji U . Antibody‐drug conjugates—an emerging class of cancer treatment. Br J Cancer. 2016;114(4):362‐367. 26742008 10.1038/bjc.2015.435 PMC4815767

[15] Khongorzul P , Ling CJ , Khan FU , Ihsan AU , Zhang J . Antibody–drug conjugates: a comprehensive review. Mol Cancer Res. 2020;18(1):3‐19. 31659006 10.1158/1541-7786.MCR-19-0582

[16] Pettinato MC . Introduction to antibody‐drug conjugates. Antibodies (Basel). 2021;10(4):42. 34842621 10.3390/antib10040042 PMC8628511

[17] Fu Z , Li S , Han S , Shi C , Zhang Y . Antibody drug conjugate: the “biological missile” for targeted cancer therapy. Signal Transduct Target Ther. 2022;7(1):93. 35318309 10.1038/s41392-022-00947-7 PMC8941077

[18] Perez HL , Cardarelli PM , Deshpande S , et al. Antibody–drug conjugates: current status and future directions. Drug Discov Today. 2014;19(7):869‐881. 24239727 10.1016/j.drudis.2013.11.004

[19] Maecker H , Jonnalagadda V , Bhakta S , Jammalamadaka V , Junutula JR . Exploration of the antibody‐drug conjugate clinical landscape. MAbs. 2023;15(1):2229101. 37639687 10.1080/19420862.2023.2229101 PMC10464553

[20] Chau CH , Steeg PS , Figg WD . Antibody–drug conjugates for cancer. Lancet. 2019;394(10200):793‐804. 31478503 10.1016/S0140-6736(19)31774-X

[21] Baah S , Laws M , Rahman KM . Antibody‐drug conjugates‐a tutorial review. Molecules. 2021;26(10):2943. 34063364 10.3390/molecules26102943 PMC8156828

[22] Lyon RP , Bovee TD , Doronina SO , et al. Reducing hydrophobicity of homogeneous antibody‐drug conjugates improves pharmacokinetics and therapeutic index. Nat Biotechnol. 2015;33(7):733‐735. 26076429 10.1038/nbt.3212

[23] Su D , Zhang D . Linker design impacts antibody‐drug conjugate pharmacokinetics and efficacy via modulating the stability and payload release efficiency. Front Pharmacol. 2021;12:687926. 34248637 10.3389/fphar.2021.687926 PMC8262647

[24] Mehrling T , Soltis D . Challenges in Optimising the successful construction of antibody drug conjugates in cancer therapy. Antibodies. 2018;7(1):11. 31544863 10.3390/antib7010011 PMC6698866

[25] Su Z , Xiao D , Xie F , et al. Antibody‐drug conjugates: recent advances in linker chemistry. Acta Pharm Sin B. 2021;11(12):3889‐3907. 35024314 10.1016/j.apsb.2021.03.042 PMC8727783

[26] Matikonda SS , McLaughlin R , Shrestha P , Lipshultz C , Schnermann MJ . Structure–activity relationships of antibody‐drug conjugates: a systematic review of chemistry on the Trastuzumab scaffold. Bioconjug Chem. 2022;33(7):1241‐1253. 35801843 10.1021/acs.bioconjchem.2c00177 PMC12974096

[27] Kalim M , Chen J , Wang S , et al. Intracellular trafficking of new anticancer therapeutics: antibody–drug conjugates. Drug des Devel Ther. 2017;11:2265‐2276. 10.2147/DDDT.S135571 PMC5546728 28814834

[28] Wang Z , Li H , Gou L , Li W , Wang Y . Antibody–drug conjugates: recent advances in payloads. Acta Pharm Sin B. 2023;13(10):4025‐4059. 37799390 10.1016/j.apsb.2023.06.015 PMC10547921

[29] Anderl J , Faulstich H , Hechler T , Kulke M . Antibody–drug conjugate payloads. In: Ducry L , ed. Antibody‐Drug Conjugates. Humana Press; 2013:51‐70. 10.1007/978-1-62703-541-5_4 23913141

[30] Birrer MJ , Moore KN , Betella I , Bates RC . Antibody‐drug conjugate‐based therapeutics: state of the science. J Natl Cancer Inst. 2019;111(6):538‐549. 30859213 10.1093/jnci/djz035

[31] Carter PJ , Senter PD . Antibody‐drug conjugates for cancer therapy. Cancer J. 2008;14(3):154‐169. 18536555 10.1097/PPO.0b013e318172d704

[32] Passaro A , Jänne PA , Peters S . Antibody‐drug conjugates in lung cancer: recent advances and implementing strategies. J Clin Oncol. 2023;41(21):3747‐3761. 37224424 10.1200/JCO.23.00013

[33] Lambert JM , Berkenblit A . Antibody–drug conjugates for cancer treatment. Annu Rev Med. 2018;69(1):191‐207. 29414262 10.1146/annurev-med-061516-121357

[34] Samantasinghar A , Sunildutt NP , Ahmed F , et al. A comprehensive review of key factors affecting the efficacy of antibody drug conjugate. Biomed Pharmacother. 2023;161:114408. 36841027 10.1016/j.biopha.2023.114408

[35] Hoffmann RM , Coumbe BGT , Josephs DH , et al. Antibody structure and engineering considerations for the design and function of antibody drug conjugates (ADCs). Onco Targets Ther. 2018;7(3):e1395127. 10.1080/2162402X.2017.1395127 PMC5769674 29375935

[36] Swaminathan M , Cortes JE . Update on the role of gemtuzumab‐ozogamicin in the treatment of acute myeloid leukemia. Ther Adv Hematol. 2023;14:20406207231154708. 36845850 10.1177/20406207231154708 PMC9943952

[37] Selby C , Yacko LR , Glode AE . Gemtuzumab ozogamicin: back again. J Adv Pract Oncol. 2019;10(1):68‐82. 31308990 PMC6605703

[38] Hills RK , Castaigne S , Appelbaum FR , et al. Addition of gemtuzumab ozogamicin to induction chemotherapy in adult patients with acute myeloid leukaemia: a meta‐analysis of individual patient data from randomised controlled trials. Lancet Oncol. 2014;15(9):986‐996. 25008258 10.1016/S1470-2045(14)70281-5 PMC4137593

[39] Lambert J , Pautas C , Terré C , et al. Gemtuzumab ozogamicin for de novo acute myeloid leukemia: final efficacy and safety updates from the open‐label, phase III ALFA‐0701 trial. Haematologica. 2019;104(1):113‐119. 30076173 10.3324/haematol.2018.188888 PMC6312010

[40] Van Der Weyden C , Dickinson M , Whisstock J , Prince HM . Brentuximab vedotin in T‐cell lymphoma. Expert Rev Hematol. 2019;12(1):5‐19. 30526166 10.1080/17474086.2019.1558399

[41] Scott LJ . Brentuximab vedotin: a review in CD30‐positive Hodgkin lymphoma. Drugs. 2017;77(4):435‐445. 28190142 10.1007/s40265-017-0705-5 PMC7102329

[42] Ansell SM . Brentuximab vedotin. Blood. 2014;124(22):3197‐3200. 25293772 10.1182/blood-2014-06-537514

[43] Straus DJ , Długosz‐Danecka M , Alekseev S , et al. Brentuximab vedotin with chemotherapy for stage III/IV classical Hodgkin lymphoma: 3‐year update of the ECHELON‐1 study. Blood. 2020;135(10):735‐742. 31945149 10.1182/blood.2019003127

[44] Ansell SM , Radford J , Connors JM , et al. Overall survival with Brentuximab Vedotin in stage III or IV Hodgkin's lymphoma. N Engl J Med. 2022;387(4):310‐320. 35830649 10.1056/NEJMoa2206125

[45] Carson KR , Newsome SD , Kim EJ , et al. Progressive multifocal leukoencephalopathy associated with brentuximab vedotin therapy: a report of 5 cases from the southern network on adverse reactions (SONAR) project. Cancer. 2014;120(16):2464‐2471. 24771533 10.1002/cncr.28712 PMC4460831

[46] Aujla A , Aujla R , Liu D . Inotuzumab ozogamicin in clinical development for acute lymphoblastic leukemia and non‐Hodgkin lymphoma. Biomark Res. 2019;7:9. 31011424 10.1186/s40364-019-0160-4 PMC6458768

[47] Savoy JM , Welch MA , Nasnas PE , Kantarjian H , Jabbour E . Inotuzumab ozogamicin for the treatment of acute lymphoblastic leukemia. Ther Adv Hematol. 2018;9(12):347‐356. 33815734 10.1177/2040620718812013 PMC7992772

[48] Uy N , Nadeau M , Stahl M , Zeidan AM . Inotuzumab ozogamicin in the treatment of relapsed/refractory acute B cell lymphoblastic leukemia. J Blood Med. 2018;9:67‐74. 29713210 10.2147/JBM.S136575 PMC5908210

[49] Kantarjian HM , DeAngelo DJ , Stelljes M , et al. Inotuzumab ozogamicin versus standard therapy for acute lymphoblastic leukemia. N Engl J Med. 2016;375(8):740‐753. 27292104 10.1056/NEJMoa1509277 PMC5594743

[50] Deeks ED . Polatuzumab vedotin: first global approval. Drugs. 2019;79(13):1467‐1475. 31352604 10.1007/s40265-019-01175-0 PMC6794237

[51] Sehn LH , Herrera AF , Flowers CR , et al. Polatuzumab vedotin in relapsed or refractory diffuse large B‐cell lymphoma. J Clin Oncol. 2020;38(2):155‐165. 31693429 10.1200/JCO.19.00172 PMC7032881

[52] Dornan D , Bennett F , Chen Y , et al. Therapeutic potential of an anti‐CD79b antibody–drug conjugate, anti‐CD79b‐vc‐MMAE, for the treatment of non‐Hodgkin lymphoma. Blood. 2009;114(13):2721‐2729. 19633198 10.1182/blood-2009-02-205500

[53] Gogia P , Ashraf H , Bhasin S , Xu Y . Antibody‐drug conjugates: a review of approved drugs and their clinical level of evidence. Cancers (Basel). 2023;15(15):3886. 37568702 10.3390/cancers15153886 PMC10417123

[54] Xu B . Loncastuximab tesirine: an effective therapy for relapsed or refractory diffuse large B‐cell lymphoma. Eur J Clin Pharmacol. 2022;78(5):707‐719. 35061047 10.1007/s00228-021-03253-3

[55] Dhillon S . Moxetumomab Pasudotox: first global approval. Drugs. 2018;78(16):1763‐1767. 30357593 10.1007/s40265-018-1000-9 PMC6323103

[56] Ketchum EB , Clarke A , Clemmons AB . Belantamab mafodotin‐BLMF: a novel antibody‐drug conjugate for treatment of patients with relapsed/refractory multiple myeloma. J Adv Pract Oncol. 2022;13(1):77‐85. 35173991 10.6004/jadpro.2022.13.1.7 PMC8805802

[57] Baines AC , Ershler R , Kanapuru B , et al. FDA approval summary: belantamab Mafodotin for patients with relapsed or refractory multiple myeloma. Clin Cancer Res. 2022;28(21):4629‐4633. 35736811 10.1158/1078-0432.CCR-22-0618 PMC9633344

[58] GlaxoSmithKline . GSK provides an update on Blenrep (belantamab mafodotin‐blmf) US marketing authorisation; 2022. Accessed January 2. 2024.

[59] Patel KC , Hageman K , Cooper MR . Ado‐trastuzumab emtansine for the treatment of human epidermal growth factor receptor 2–positive metastatic breast cancer. Am J Health‐Syst Pharm. 2014;71(7):537‐548. 24644113 10.2146/ajhp130342

[60] Corrigan PA , Cicci TA , Auten JJ , Lowe DK . Ado‐trastuzumab emtansine:a HER2‐positive targeted antibody‐drug conjugate. Ann Pharmacother. 2014;48(11):1484‐1493. 25082874 10.1177/1060028014545354

[61] Lewis Phillips GD , Li G , Dugger DL , et al. Targeting HER2‐positive breast cancer with trastuzumab‐DM1, an antibody–cytotoxic drug conjugate. Cancer Res. 2008;68(22):9280‐9290. 19010901 10.1158/0008-5472.CAN-08-1776

[62] Hunter FW , Barker HR , Lipert B , et al. Mechanisms of resistance to trastuzumab emtansine (T‐DM1) in HER2‐positive breast cancer. Br J Cancer. 2020;122(5):603‐612. 31839676 10.1038/s41416-019-0635-y PMC7054312

[63] Verma S , Miles D , Gianni L , et al. Trastuzumab emtansine for HER2‐positive advanced breast cancer. N Engl J Med. 2012;367(19):1783‐1791. 23020162 10.1056/NEJMoa1209124 PMC5125250

[64] Gouda MA , Subbiah V . Strategies for mitigating antibody‐drug conjugate related adverse events for precision therapy. The Cancer Journal. 2022;28(6):496‐507. 36383913 10.1097/PPO.0000000000000627 PMC11874066

[65] Mantia CM , Sonpavde G . Enfortumab vedotin‐ejfv for the treatment of advanced urothelial carcinoma. Expert Rev Anticancer Ther. 2022;22(5):449‐455. 35466857 10.1080/14737140.2022.2069563

[66] Chang E , Weinstock C , Zhang L , et al. FDA approval summary: enfortumab vedotin for locally advanced or metastatic urothelial carcinoma. Clin Cancer Res. 2021;27(4):922‐927. 32962979 10.1158/1078-0432.CCR-20-2275

[67] Halford Z , Anderson MK , Clark MD . Enfortumab vedotin‐ejfv: a first‐in‐class anti–nectin‐4 antibody‐drug conjugate for the management of urothelial carcinoma. Annals of Pharmacotherapy. 2021;55(6):772‐782. 32945172 10.1177/1060028020960402

[68] Narayan P , Osgood CL , Singh H , et al. FDA approval summary: fam‐trastuzumab deruxtecan‐Nxki for the treatment of unresectable or metastatic HER2‐positive breast cancer. Clin Cancer Res. 2021;27(16):4478‐4485. 33753456 10.1158/1078-0432.CCR-20-4557 PMC8570919

[69] Nguyen X , Hooper M , Borlagdan JP , Palumbo A . A review of fam‐trastuzumab deruxtecan‐nxki in HER2‐positive breast cancer. Ann Pharmacother. 2021;55(11):1410‐1418. 33629601 10.1177/1060028021998320

[70] Narayan P , Dilawari A , Osgood C , et al. US Food and Drug Administration approval summary: fam‐Trastuzumab Deruxtecan‐nxki for human epidermal growth factor receptor 2‐low Unresectable or metastatic breast cancer. J Clin Oncol. 2023;41(11):2108‐2116. 36780610 10.1200/JCO.22.02447

[71] Li BT , Smit EF , Goto Y , et al. Trastuzumab deruxtecan in HER2‐mutant non‐small‐cell lung cancer. N Engl J Med. 2022;386(3):241‐251. 34534430 10.1056/NEJMoa2112431 PMC9066448

[72] Syed YY . Sacituzumab govitecan: first approval. Drugs. 2020;80(10):1019‐1025. 32529410 10.1007/s40265-020-01337-5 PMC7288263

[73] Rugo HS , Bardia A , Marmé F , et al. Overall survival with sacituzumab govitecan in hormone receptor‐positive and human epidermal growth factor receptor 2‐negative metastatic breast cancer (TROPiCS‐02): a randomised, open‐label, multicentre, phase 3 trial. Lancet. 2023;402(10411):1423‐1433. 37633306 10.1016/S0140-6736(23)01245-X

[74] Heitz N , Greer SC , Halford Z . A review of tisotumab Vedotin‐tftv in recurrent or metastatic cervical cancer. Ann Pharmacother. 2023;57(5):585‐596. 35962528 10.1177/10600280221118370

[75] Coleman RL , Lorusso D , Gennigens C , et al. Efficacy and safety of tisotumab vedotin in previously treated recurrent or metastatic cervical cancer (innovaTV 204/GOG‐3023/ENGOT‐cx6): a multicentre, open‐label, single‐arm, phase 2 study. Lancet Oncol. 2021;22(5):609‐619. 33845034 10.1016/S1470-2045(21)00056-5

[76] Aschenbrenner DS . New drug treats cervical cancer. Am J Nurs. 2022;122(1):21. 10.1097/01.NAJ.0000815420.91630.18 34941590

[77] Dilawari A , Shah M , Ison G , et al. FDA approval summary: mirvetuximab soravtansine‐Gynx for FRα‐positive, platinum‐resistant ovarian cancer. Clin Cancer Res. 2023;29(19):3835‐3840. 37212825 10.1158/1078-0432.CCR-23-0991 PMC10592645

[78] Moore KN , Angelergues A , Konecny GE , et al. Mirvetuximab soravtansine in FRα‐positive, platinum‐resistant ovarian cancer. N Engl J Med. 2023;389(23):2162‐2174. 38055253 10.1056/NEJMoa2309169

[79] Ponziani S , Di Vittorio G , Pitari G , et al. Antibody‐drug conjugates: the new frontier of chemotherapy. Int J Mol Sci. 2020;21(15):5510. 32752132 10.3390/ijms21155510 PMC7432430

[80] Damelin M , Zhong W , Myers J , Sapra P . Evolving strategies for target selection for antibody‐drug conjugates. Pharm Res. 2015;32(11):3494‐3507. 25585957 10.1007/s11095-015-1624-3

[81] Singh S , Serwer L , DuPage A , et al. Nonclinical efficacy and safety of CX‐2029, an anti‐CD71 Probody‐drug conjugate. Mol Cancer Ther. 2022;21(8):1326‐1336. 35666803 10.1158/1535-7163.MCT-21-0193 PMC9662867

[82] Criscitiello C , Morganti S , Curigliano G . Antibody–drug conjugates in solid tumors: a look into novel targets. J Hematol Oncol. 2021;14(1):20. 33509252 10.1186/s13045-021-01035-z PMC7844898

[83] Drago JZ , Modi S , Chandarlapaty S . Unlocking the potential of antibody–drug conjugates for cancer therapy. Nat Rev Clin Oncol. 2021;18(6):327‐344. 33558752 10.1038/s41571-021-00470-8 PMC8287784

[84] Conilh L , Sadilkova L , Viricel W , Dumontet C . Payload diversification: a key step in the development of antibody–drug conjugates. J Hematol Oncol. 2023;16(1):3. 36650546 10.1186/s13045-022-01397-y PMC9847035

[85] Strop P , Delaria K , Foletti D , et al. Site‐specific conjugation improves therapeutic index of antibody drug conjugates with high drug loading. Nat Biotechnol. 2015;33(7):694‐696. 26154005 10.1038/nbt.3274

[86] Sun X , Ponte JF , Yoder NC , et al. Effects of drug–antibody ratio on pharmacokinetics, biodistribution, efficacy, and tolerability of antibody–Maytansinoid conjugates. Bioconjug Chem. 2017;28(5):1371‐1381. 28388844 10.1021/acs.bioconjchem.7b00062

[87] Hamblett KJ , Senter PD , Chace DF , et al. Effects of drug loading on the antitumor activity of a monoclonal antibody drug conjugate. Clin Cancer Res. 2004;10(20):7063‐7070. 15501986 10.1158/1078-0432.CCR-04-0789

[88] Duerr C , Friess W . Antibody‐drug conjugates‐stability and formulation. Eur J Pharm Biopharm. 2019;139:168‐176. 30940541 10.1016/j.ejpb.2019.03.021

[89] Sochaj AM , Świderska KW , Otlewski J . Current methods for the synthesis of homogeneous antibody‐drug conjugates. Biotechnol Adv. 2015;33(6 Pt 1):775‐784. 25981886 10.1016/j.biotechadv.2015.05.001

[90] Bodyak ND , Mosher R , Yurkovetskiy AV , et al. The dolaflexin‐based antibody–drug conjugate XMT‐1536 targets the solid tumor lineage antigen SLC34A2/NaPi2b. Mol Cancer Ther. 2021;20(5):896‐905. 33722858 10.1158/1535-7163.MCT-20-0183

[91] Junutula JR , Raab H , Clark S , et al. Site‐specific conjugation of a cytotoxic drug to an antibody improves the therapeutic index. Nat Biotechnol. 2008;26(8):925‐932. 18641636 10.1038/nbt.1480

[92] Strop P , Liu S‐H , Dorywalska M , et al. Location matters: site of conjugation modulates stability and pharmacokinetics of antibody drug conjugates. Chem Biol. 2013;20(2):161‐167. 23438745 10.1016/j.chembiol.2013.01.010

[93] Adem YT , Schwarz KA , Duenas E , Patapoff TW , Galush WJ , Esue O . Auristatin antibody drug conjugate physical instability and the role of drug payload. Bioconjug Chem. 2014;25(4):656‐664. 24559399 10.1021/bc400439x

[94] Shen B‐Q , Xu K , Liu L , et al. Conjugation site modulates the in vivo stability and therapeutic activity of antibody‐drug conjugates. Nat Biotechnol. 2012;30(2):184‐189. 22267010 10.1038/nbt.2108

[95] Junutula JR , Flagella KM , Graham RA , et al. Engineered thio‐trastuzumab‐DM1 conjugate with an improved therapeutic index to target human epidermal growth factor receptor 2–positive breast cancer. Clin Cancer Res. 2010;16(19):4769‐4778. 20805300 10.1158/1078-0432.CCR-10-0987

[96] Cheng Y , Yuan X , Tian Q , et al. Preclinical profiles of SKB264, a novel anti‐TROP2 antibody conjugated to topoisomerase inhibitor, demonstrated promising antitumor efficacy compared to IMMU‐132. Front Oncol. 2022;12:951589. 36620535 10.3389/fonc.2022.951589 PMC9817100

[97] Li L , Hau A , Tong W , et al. Abstract LB‐227: preclinical development and characterization of STI‐6129, an anti‐CD38 antibody‐drug conjugate, as a new therapeutic agent for multiple myeloma. Cancer Res. 2020;80(16_Supplement):LB227.

[98] Ceci C , Lacal PM , Graziani G . Antibody‐drug conjugates: resurgent anticancer agents with multi‐targeted therapeutic potential. Pharmacol Ther. 2022;236:108106. 34990642 10.1016/j.pharmthera.2021.108106

[99] Adams S , Wilhelm A , Harvey L , et al. IMGN632: a CD123‐targeting antibody‐drug conjugate (ADC) with a novel DNA‐alkylating payload, is highly active and prolongs survival in acute myeloid leukemia (AML) xenograft models. Blood. 2016;128(22):2832.

[100] Walsh SJ , Bargh JD , Dannheim FM , et al. Site‐selective modification strategies in antibody–drug conjugates. Chem Soc Rev. 2021;50(2):1305‐1353. 33290462 10.1039/d0cs00310g

[101] Beck A , Goetsch L , Dumontet C , Corvaïa N . Strategies and challenges for the next generation of antibody–drug conjugates. Nat Rev Drug Discov. 2017;16(5):315‐337. 28303026 10.1038/nrd.2016.268

[102] Yoder NC , Bai C , Tavares D , et al. A case study comparing heterogeneous lysine‐ and site‐specific cysteine‐conjugated maytansinoid antibody‐drug conjugates (ADCs) illustrates the benefits of lysine conjugation. Mol Pharm. 2019;16(9):3926‐3937. 31287952 10.1021/acs.molpharmaceut.9b00529

[103] Skidmore L , Sakamuri S , Knudsen NA , et al. ARX788, a site‐specific anti‐HER2 antibody‐drug conjugate, demonstrates potent and selective activity in HER2‐low and T‐DM1‐resistant breast and gastric cancers. Mol Cancer Ther. 2020;19(9):1833‐1843. 32669315 10.1158/1535-7163.MCT-19-1004

[104] Wijdeven MA , van Geel R , Hoogenboom JH , et al. Enzymatic glycan remodeling‐metal free click (GlycoConnect™) provides homogenous antibody‐drug conjugates with improved stability and therapeutic index without sequence engineering. MAbs. 2022;14(1):2078466. 35634725 10.1080/19420862.2022.2078466 PMC9154768

[105] Zammarchi F , Corbett S , Adams L , et al. hLL2‐Cys‐PBD, a new site‐specifically conjugated, Pyrrolobenzodiazepine (PBD) dimer‐based antibody drug conjugate (ADC) targeting CD22‐expressing B‐cell malignancies. Blood. 2016;128(22):4176.

[106] Khoury R , Saleh K , Khalife N , et al. Mechanisms of resistance to antibody‐drug conjugates. Int J Mol Sci. 2023;24(11):9674. 37298631 10.3390/ijms24119674 PMC10253543

[107] Loganzo F , Sung M , Gerber H‐P . Mechanisms of resistance to antibody–drug conjugates. Mol Cancer Ther. 2016;15(12):2825‐2834. 27780876 10.1158/1535-7163.MCT-16-0408

[108] Chang HL , Schwettmann B , McArthur HL , Chan IS . Antibody‐drug conjugates in breast cancer: overcoming resistance and boosting immune response. J Clin Invest. 2023;133(18):e172156. 37712425 10.1172/JCI172156 PMC10503805

[109] Loganzo F , Tan X , Sung M , et al. Tumor cells chronically treated with a trastuzumab‐maytansinoid antibody‐drug conjugate develop varied resistance mechanisms but respond to alternate treatments. Mol Cancer Ther. 2015;14(4):952‐963. 25646013 10.1158/1535-7163.MCT-14-0862

[110] Chen R , Hou J , Newman E , et al. CD30 downregulation, MMAE resistance, and MDR1 upregulation are all associated with resistance to Brentuximab Vedotin. Mol Cancer Ther. 2015;14(6):1376‐1384. 25840583 10.1158/1535-7163.MCT-15-0036 PMC4458438

[111] Filho OM , Viale G , Stein S , et al. Impact of HER2 heterogeneity on treatment response of early‐stage HER2‐positive breast cancer: phase II Neoadjuvant clinical trial of T‐DM1 combined with Pertuzumab. Cancer Discov. 2021;11(10):2474‐2487. 33941592 10.1158/2159-8290.CD-20-1557 PMC8598376

[112] Gebhart G , Lamberts LE , Wimana Z , et al. Molecular imaging as a tool to investigate heterogeneity of advanced HER2‐positive breast cancer and to predict patient outcome under trastuzumab emtansine (T‐DM1): the ZEPHIR trial. Ann Oncol. 2016;27(4):619‐624. 26598545 10.1093/annonc/mdv577

[113] Coates JT , Sun S , Leshchiner I , et al. Parallel genomic alterations of antigen and payload targets mediate polyclonal acquired clinical resistance to sacituzumab govitecan in triple‐negative breast cancer. Cancer Discov. 2021;11(10):2436‐2445. 34404686 10.1158/2159-8290.CD-21-0702 PMC8495771

[114] Ogitani Y , Aida T , Hagihara K , et al. DS‐8201a, a novel HER2‐targeting ADC with a novel DNA topoisomerase I inhibitor, demonstrates a promising antitumor efficacy with differentiation from T‐DM1. Clin Cancer Res. 2016;22(20):5097‐5108. 27026201 10.1158/1078-0432.CCR-15-2822

[115] Takegawa N , Nonagase Y , Yonesaka K , et al. DS‐8201a, a new HER2‐targeting antibody‐drug conjugate incorporating a novel DNA topoisomerase I inhibitor, overcomes HER2‐positive gastric cancer T‐DM1 resistance. Int J Cancer. 2017;141(8):1682‐1689. 28677116 10.1002/ijc.30870

[116] Yu SF , Zheng B , Go M , et al. A novel anti‐CD22 Anthracycline‐based antibody‐drug conjugate (ADC) that overcomes resistance to auristatin‐based ADCs. Clin Cancer Res. 2015;21(14):3298‐3306. 25840969 10.1158/1078-0432.CCR-14-2035

[117] García‐Alonso S , Ocaña A , Pandiella A . Resistance to antibody–drug conjugates. Cancer Res. 2018;78(9):2159‐2165. 29653942 10.1158/0008-5472.CAN-17-3671

[118] Kovtun YV , Audette CA , Mayo MF , et al. Antibody‐maytansinoid conjugates designed to bypass multidrug resistance. Cancer Res. 2010;70(6):2528‐2537. 20197459 10.1158/0008-5472.CAN-09-3546

[119] Sung M , Tan X , Lu B , et al. Caveolae‐mediated endocytosis as a novel mechanism of resistance to trastuzumab emtansine (T‐DM1). Mol Cancer Ther. 2018;17(1):243‐253. 29054985 10.1158/1535-7163.MCT-17-0403

[120] Ríos‐Luci C , García‐Alonso S , Díaz‐Rodríguez E , et al. Resistance to the antibody–drug conjugate T‐DM1 is based in a reduction in lysosomal proteolytic activity. Cancer Res. 2017;77(17):4639‐4651. 28687619 10.1158/0008-5472.CAN-16-3127

[121] Tumey LN . An overview of the current ADC discovery landscape. In: Tumey LN , ed. Antibody‐Drug Conjugates: Methods and Protocols. Springer; 2020:1‐22. 10.1007/978-1-4939-9929-3_1 31643046

[122] Wang H , Wang W , Xu Y , et al. Aberrant intracellular metabolism of T‐DM1 confers T‐DM1 resistance in human epidermal growth factor receptor 2‐positive gastric cancer cells. Cancer Sci. 2017;108(7):1458‐1468. 28388007 10.1111/cas.13253 PMC5497802

[123] Trudeau KM , Colby AH , Zeng J , et al. Lysosome acidification by photoactivated nanoparticles restores autophagy under lipotoxicity. J Cell Biol. 2016;214(1):25‐34. 27377248 10.1083/jcb.201511042 PMC4932370

[124] Singh D , Dheer D , Samykutty A , Shankar R . Antibody drug conjugates in gastrointestinal cancer: from lab to clinical development. J Control Release. 2021;340:1‐34. 34673122 10.1016/j.jconrel.2021.10.006

[125] Trail PA , Dubowchik GM , Lowinger TB . Antibody drug conjugates for treatment of breast cancer: novel targets and diverse approaches in ADC design. Pharmacol Ther. 2018;181:126‐142. 28757155 10.1016/j.pharmthera.2017.07.013

[126] Xu Z , Guo D , Jiang Z , et al. Novel HER2‐targeting antibody‐drug conjugates of Trastuzumab beyond T‐DM1 in breast cancer: trastuzumab deruxtecan(DS‐8201a) and (Vic‐)trastuzumab duocarmazine (SYD985). Eur J Med Chem. 2019;183:111682. 31563805 10.1016/j.ejmech.2019.111682

[127] Knuehl C , Toleikis L , Dotterweich J , et al. Abstract 5284: M1231 is a bispecific anti‐MUC1xEGFR antibody‐drug conjugate designed to treat solid tumors with MUC1 and EGFR co‐expression. Cancer Res. 2022;82(12_Supplement):5284.

[128] Zutshi A , Neuteboom B , Kumar S , et al. Abstract 5423: translational PK/PD/efficacy modeling and efficacious human dose prediction for a first‐in‐class MUC1‐EGFR (M1231) bispecific antibody drug conjugate. Cancer Res. 2022;82(12_Supplement):5423.

[129] Li JY , Perry SR , Muniz‐Medina V , et al. A Biparatopic HER2‐targeting antibody‐drug conjugate induces tumor regression in primary models refractory to or ineligible for HER2‐targeted therapy. Cancer Cell. 2016;29(1):117‐129. 26766593 10.1016/j.ccell.2015.12.008

[130] Weisser NE , Sanches M , Escobar‐Cabrera E , et al. An anti‐HER2 biparatopic antibody that induces unique HER2 clustering and complement‐dependent cytotoxicity. Nat Commun. 2023;14(1):1394. 36914633 10.1038/s41467-023-37029-3 PMC10011572

[131] DaSilva JO , Yang K , Surriga O , et al. A Biparatopic antibody‐drug conjugate to treat MET‐expressing cancers, including those that are unresponsive to MET pathway blockade. Mol Cancer Ther. 2021;20(10):1966‐1976. 34315762 10.1158/1535-7163.MCT-21-0009 PMC9398133

[132] Kast F , Schwill M , Stüber JC , et al. Engineering an anti‐HER2 biparatopic antibody with a multimodal mechanism of action. Nat Commun. 2021;12(1):3790. 34145240 10.1038/s41467-021-23948-6 PMC8213836

[133] Spangler JB , Neil JR , Abramovitch S , et al. Combination antibody treatment down‐regulates epidermal growth factor receptor by inhibiting endosomal recycling. Proc Natl Acad Sci U S A. 2010;107(30):13252‐13257. 20616078 10.1073/pnas.0913476107 PMC2922117

[134] Pegram MD , Hamilton EP , Tan AR , et al. First‐in‐human, phase 1 dose‐escalation study of biparatopic anti‐HER2 antibody‐drug conjugate MEDI4276 in patients with HER2‐positive advanced breast or gastric cancer. Mol Cancer Ther. 2021;20(8):1442‐1453. 34045233 10.1158/1535-7163.MCT-20-0014 PMC9398097

[135] Fan J , Zhuang X , Yang X , et al. A multivalent biparatopic EGFR‐targeting nanobody drug conjugate displays potent anticancer activity in solid tumor models. Signal Transduct Target Ther. 2021;6(1):320. 34475375 10.1038/s41392-021-00666-5 PMC8413295

[136] Nervig CS , Owen SC . Advances in the development of dual‐drug antibody drug conjugates. ADC Rev J Antibody Drug Conjug. 2023. doi:10.14229/jadc.2023.01.05.001

[137] Levengood MR , Zhang X , Hunter JH , et al. Orthogonal cysteine protection enables homogeneous multi‐drug antibody‐drug conjugates. Angew Chem Int Ed Engl. 2017;56(3):733‐737. 27966822 10.1002/anie.201608292 PMC5299463

[138] Yamazaki CM , Yamaguchi A , Anami Y , et al. Antibody‐drug conjugates with dual payloads for combating breast tumor heterogeneity and drug resistance. Nat Commun. 2021;12(1):3528. 34112795 10.1038/s41467-021-23793-7 PMC8192907

[139] Kumar A , Kinneer K , Masterson L , et al. Synthesis of a heterotrifunctional linker for the site‐specific preparation of antibody‐drug conjugates with two distinct warheads. Bioorg Med Chem Lett. 2018;28(23):3617‐3621. 30389292 10.1016/j.bmcl.2018.10.043

